# Intercoupled electrocatalytic ammonia synthesis via a looped Li–N_2_/H_2_ battery

**DOI:** 10.1093/nsr/nwaf586

**Published:** 2025-12-27

**Authors:** Zhendong Wang, Xiang Zhang, Zhiwei Xiao, Qian Feng, Jing Lin, Linlin Zhang, Yangyang Feng, Yaobing Wang

**Affiliations:** College of Chemistry and Materials Science, Fujian Normal University, Fuzhou 350007, China; CAS Key Laboratory of Design and Assembly of Functional Nanostructures, and Fujian Provincial Key Laboratory of Nanomaterials, State Key Laboratory of Structural Chemistry, Fujian Institute of Research on the Structure of Matter, Chinese Academy of Sciences, Fuzhou 350002, China; Fujian College, University of Chinese Academy of Sciences, Fuzhou 350002, China; CAS Key Laboratory of Design and Assembly of Functional Nanostructures, and Fujian Provincial Key Laboratory of Nanomaterials, State Key Laboratory of Structural Chemistry, Fujian Institute of Research on the Structure of Matter, Chinese Academy of Sciences, Fuzhou 350002, China; University of Chinese Academy of Sciences, Beijing 100049, China; Fujian Science and Technology Innovation Laboratory for Optoelectronic Information of China, Fuzhou 350108, China; CAS Key Laboratory of Design and Assembly of Functional Nanostructures, and Fujian Provincial Key Laboratory of Nanomaterials, State Key Laboratory of Structural Chemistry, Fujian Institute of Research on the Structure of Matter, Chinese Academy of Sciences, Fuzhou 350002, China; University of Chinese Academy of Sciences, Beijing 100049, China; Fujian College, University of Chinese Academy of Sciences, Fuzhou 350002, China; CAS Key Laboratory of Design and Assembly of Functional Nanostructures, and Fujian Provincial Key Laboratory of Nanomaterials, State Key Laboratory of Structural Chemistry, Fujian Institute of Research on the Structure of Matter, Chinese Academy of Sciences, Fuzhou 350002, China; Fujian College, University of Chinese Academy of Sciences, Fuzhou 350002, China; CAS Key Laboratory of Design and Assembly of Functional Nanostructures, and Fujian Provincial Key Laboratory of Nanomaterials, State Key Laboratory of Structural Chemistry, Fujian Institute of Research on the Structure of Matter, Chinese Academy of Sciences, Fuzhou 350002, China; CAS Key Laboratory of Design and Assembly of Functional Nanostructures, and Fujian Provincial Key Laboratory of Nanomaterials, State Key Laboratory of Structural Chemistry, Fujian Institute of Research on the Structure of Matter, Chinese Academy of Sciences, Fuzhou 350002, China; CAS Key Laboratory of Design and Assembly of Functional Nanostructures, and Fujian Provincial Key Laboratory of Nanomaterials, State Key Laboratory of Structural Chemistry, Fujian Institute of Research on the Structure of Matter, Chinese Academy of Sciences, Fuzhou 350002, China; University of Chinese Academy of Sciences, Beijing 100049, China; CAS Key Laboratory of Design and Assembly of Functional Nanostructures, and Fujian Provincial Key Laboratory of Nanomaterials, State Key Laboratory of Structural Chemistry, Fujian Institute of Research on the Structure of Matter, Chinese Academy of Sciences, Fuzhou 350002, China; University of Chinese Academy of Sciences, Beijing 100049, China; Fujian Science and Technology Innovation Laboratory for Optoelectronic Information of China, Fuzhou 350108, China

**Keywords:** decoupled NH_3_ electrosynthesis, looped Li–N_2_/H_2_ battery, electrocatalytic N_2_ reduction, electrocatalytic H_2_ oxidation, bifunctional electrocatalyst

## Abstract

Electrochemical ammonia (NH_3_) synthesis offers a sustainable pathway for the chemical industry. However, the fundamental proton-coupled nitrogen (N_2_) reduction process has led to the competing H_2_ evolution and low energy efficiency, particularly at high current densities. Herein, we present the design of a looped Li–N_2_/H_2_ battery that decouples N_2_ reduction from protonation by two separate sub-reactions of electrocatalytic N_2_ reduction in discharging (6Li^+^ + 6e^−^ + N_2_ → 2Li_3_N) and electrocatalytic H_2_ oxidation in charging (H_2_ → 2H^+^ + 2e^−^), which are intercoupled into a synthetic loop to enable NH_3_ synthesis (Li_3_N + 3H^+^ → NH_3_ + 3Li^+^) without H_2_ evolution. This approach achieves record-high energy efficiency (26.0% ± 0.9%), Faradaic efficiency (63.7% ± 2.3%), and high NH_3_ production rate (1 mA cm^−2^, 0.12 mol h^−1^ m^−2^) under mild conditions. These results significantly lower the cost of ammonia production compared to conventional electrochemical methods, highlighting its promising potential for practical applications.

## Teaser

This work reports a looped Li-N2/H2 battery featuring a proton-decoupled N2 electrocatalytic mechanism, which significantly boosts electrochemical NH3 productivity and overall device performances.

## INTRODUCTION

**Figure 1.**
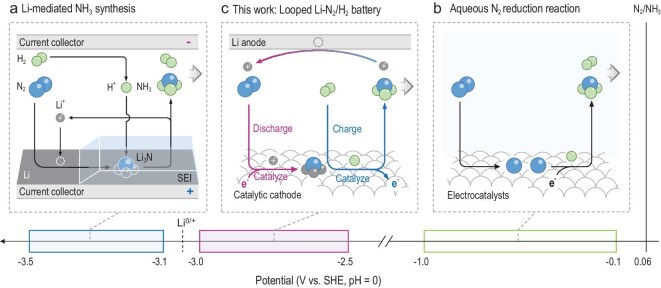
Schematic comparison of N_2_ reduction methods at different overpotentials. (a) Li-mediated NH_3_ synthesis with an overpotential of >3 V at the cathode. (b) Aqueous N_2_ reduction method within an overpotential range of 0.1–1 V. (c) This work: looped Li–N_2_/H_2_ battery within an overpotential range of 2–3 V at the cathode.

Ammonia (NH_3_) is crucial for fertilizers and chemicals, and holds promise as a carbon-free energy source [1]. Thermal catalytic synthesis of NH_3_ from N_2_ and H_2_, known as the Haber–Bosch (H–B) process, is the most prevalent method for large-scale ammonia synthesis, despite the need for harsh conditions (400–500°C, 150–200 atm) for N≡N activation [1–4]. Inspired by the nitrogenase in nature for biological NH_3_ production through multiple proton and electron transfer steps, electrochemical NH_3_ synthesis from N_2_ and H_2_O via proton-coupled N_2_ reduction offers a carbon-neutral alternative by using electrical overpotential (∼0.3 V versus standard hydrogen electrode, SHE) instead of high heat for milder N_2_ activation and reduction (Fig. 1) [4–6]. Unfortunately, H_2_ evolution has long been the most competitive reaction in NH_3_ production [2], due to not only the poor solubility of N_2_, but also the more favorable kinetics of H_2_ evolution than those of N_2_ reduction and protonation [7,8], leading to low NH_3_ current density (*J*_NH3_) and yields, even when using electrocatalysts with intrinsic H_2_ evolution inertness or restricted proton/electron accessibility [9]. In contrast, non-aqueous lithium-mediated N_2_ reduction (LiNR), which involves the hydrogenation of Li_3_N resulting from thermochemical N_2_ reduction with electrochemically deposited metallic Li [5], achieves NH_3_ synthesis at a high Faradaic efficiency (*FE*_NH3_) and *J*_NH3_ but at the cost of a large overpotential (>3 V) and low energy efficiency [10–13]. Moreover, this method requires the collection of electrons and protons on the same electrode (similar to water systems), potentially leading to unwanted H_2_ generation through Li–H bond formation [12]. Therefore, decoupling the roles of electrons and protons, i.e. allowing electrons to participate solely in the N_2_ reduction without interfering with the subsequent protonation process, is crucial for electrochemical NH_3_ synthesis without H_2_ evolution. While an Li-cycling strategy and Li–N_2_ batteries have been reported to achieve this goal, the associated costs of Li retrieval under harsh conditions (*E* = 2.8 V vs Li/Li^+^, 427°C) or the need to disassemble the battery have hindered their sustainability and economic viabilities [2,4,14–16].

Building on these efforts, we present here a looped Li–N_2_/H_2_ battery that enables electrochemical NH_3_ synthesis via a proton-decoupled electrocatalytic N_2_ reduction mechanism (Fig. 1). This strategy couples the discharge reaction of electrocatalytic N_2_ reduction to Li_3_N with the charge reaction of electrocatalytic H_2_ oxidation, achieving simultaneous NH_3_ production and Li retrieval (2Li_3_N + 3H_2_ → 2NH_3_ + 6Li) in a looped manner. Distinguished from conventional nitrogen reduction reaction (NRR) and LiNR methods, this approach temporally separates N_2_ reduction from protonation while applying an oxidative bias during Li_3_N protonation, thereby avoiding the competing H_2_ evolution. In addition, this innovation enables NH_3_ synthesis at a moderate overpotential (N_2_ + 6Li^+^ + 6e^−^ → 2Li_3_N; *E*^0^ = −2.60 V vs. SHE) [17], corresponding to a theoretical energy efficiency of up to 47%. Moreover, the overall synthetic loop is thermodynamically favorable (Δ*G* = −32.90 kJ mol^−1^), providing surplus electricity to the system. By completing the discharge–charge loop, the electrochemical NH_3_ synthesis in the Li–N_2_ battery transforms from an otherwise unsustainable process into one that is sustainable and continuous, particularly advantageous for small-scale on-demand NH_3_ production, and offers compatibility with other technologies, including hydrogen storage, electricity storage and conversion.

To enable the Li–N_2_/H_2_ battery, it is crucial to have a catalytic cathode with high activity for both electrocatalytic N_2_ reduction and the H_2_ oxidation reaction (HOR). Platinum (Pt), known for its excellent electrocatalytic HOR activity [18], also shows promise for aqueous [19–21] and non-aqueous [22] N_2_ activation and reduction. Herein, we employed a Pt-based bifunctional cathode with electrocatalytic activities for both N_2_-to-Li_3_N reduction (63.7% ± 2.3% FE) and HOR (99% FE), and the resulting looped Li–N_2_/H_2_ battery achieved a maximum EE of 26.0% ± 0.9%, a *J*_NH3_ of up to 1 mA cm^−2^, and an NH_3_ yield rate (*R*_NH3_) of 3.5 nmol s^−1^ mA^−1^ (0.12 mol h^−^^1^ m^−2^). These performance metrics are comparable to established electrochemical approaches, and demonstrate the lowest estimated NH_3_ production cost. By cascading the two catalytic reactions of N_2_ reduction and H_2_ oxidation, the presented Li–N_2_/H_2_ battery fills the technical gap in electrochemical NH_3_ production in the 2.0–3.0 V overpotential region, contributing to the practical industrialization of electrochemical NH_3_ synthesis.

## RESULTS

### Electrochemical N_2_ reduction to Li_3_N

**Figure 2.**
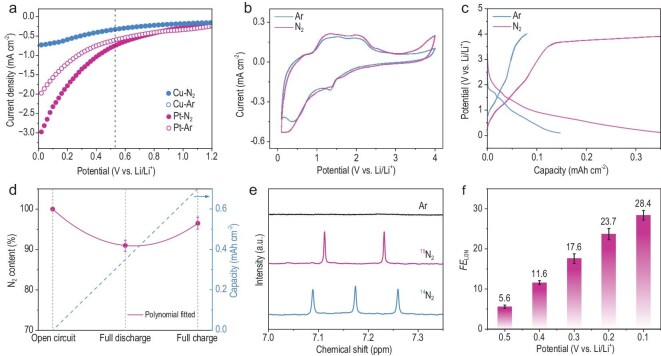
Electrocatalytic N_2_ reduction to Li_3_N. (a) LSV, (b) CV and (c) GCD profiles of Pt in an Ar and N_2_ atmosphere. (d) Evolution of the N_2_ content from open circuit potential to fully discharged and charged state. ‘N_2_ content’ refers specifically to the absolute amount of N_2_ (in moles) in the closed cell. (e) Isotope-labeled controls using ^15^N_2_. (f) Value of *FE*_Li3N_ at different cut-off potentials and ambient conditions.

All experiments in this study were conducted within a high-purity argon (Ar)-filled glove box to eliminate any potential contamination from gases like NO_x_, H_2_O and O_2_ in the gaseous reactant (N_2_, H_2_). For the electrocatalytic N_2_ reduction, a Pt mesh served as the working electrode, and its activity was initially assessed in a three-electrode system with Ag as the reference electrode and Pt foil as the counter electrode in a 1.0 M lithium bis(trifluoromethylsulfonimide) (LiTFSI)/tetraethylene glycol dimethyl ether (TEGDME) electrolyte. Linear scanning voltammetry (LSV) of Pt showed increased current densities in an N_2_ atmosphere compared to Ar, indicating the occurrence of NRRs within the potential range of 0.5 to 0.05 V vs. Li/Li^+^ (Fig. 2a). In contrast, copper (Cu), employed as the control sample, consistently exhibited similar current densities across different atmospheres. This highlights the pivotal influence of electrode selection in determining whether NRRs occur, providing further confirmation of the catalytic nature of the process. Cyclic voltammetry (CV) was then conducted in an Li–N_2_ battery system, where Pt acted as the cathode and Li foil as the anode. Under an Ar atmosphere, redox peaks were observed at 0.5 and 1.5 V, indicating the reversible alloying of Pt with Li (Fig. 2b). Transitioning to an N_2_ atmosphere led to additional peaks at 0.4 and 3.8 V, along with flat plateaus in the discharge (∼0.5 V) and charge (∼3.8 V) in galvanostatic charge–discharge (GCD) measurements (Fig. 2c). The presence of stable voltage platforms during cycling tests confirmed the good reversibility (Fig. S1).

The products of the discharge and charge reaction were identified. Prior to characterization, all samples were cleaned with dried tetrahydrofuran to remove the electrolyte. Energy-dispersive spectrometry (EDS) images revealed a substantial amount of nitrogen fixed on the Pt surface after discharging to 0.1 V (with an N content of 10.4 at.%; Fig. S2), while only traces of nitrogen were observed after the subsequent charging process (with an N content of 0.8 at.%). The nitrogen that had been fixed was confirmed to be Li_3_N through X-ray diffraction patterns and N1s spectra obtained in X-ray photoelectron spectroscopy [15,16]. Gas chromatography (GC) analysis indicated that the overall N_2_ content initially decreased, with a decrease in discharge voltage, and then increased following full charging. However, it did not fully return to its initial state (Fig. 2d). This suggests the possibility of irreversible reactions, such as the formation of LiNO_x_, likely due to the presence of trace O_2_. This observation correlates with the findings from EDS mapping and N1s spectra.

The Faradaic efficiency of the electrocatalytic N_2_ reduction to Li_3_N (*FE*_Li3N_) was quantified by discharging the battery to different cut-off voltages. Following discharge, the Pt electrode was immersed in water to obtain soluble NH_3_ through Li_3_N hydrolysis. To ensure the reliability of the evidence regarding N_2_ electrocatalytic reduction performance, we followed the experimental protocol outlined in Fig. S3. The quantity of NH_3_ derived from Li_3_N consistently increased as the cut-off potentials decreased, as confirmed by both ^1^H nuclear magnetic resonance (NMR) and colorimetric methods (Figs S4–S6). The amount of Li_3_N increased with decreasing cut-off voltages, with the highest *FE*_Li3N_ reaching 28.4% at 0.1 V vs. Li/Li^+^. ^15^N isotope labelling experiments, along with control experiments conducted under an Ar atmosphere, were undertaken to confirm that the feedstock N_2_ exclusively served as the nitrogen source for Li_3_N production (Fig. 2e and Fig. S6). The *FE*_Li3N_ values were similar across different electrolytes (LiTFSI, LiPF_6_ and LiClO_4_), indicating that the nitrogen source in Li_3_N was not from LiTFSI (Fig. S7). Given that the deposition of metallic Li at the negative electrode at 0.1 V is thermodynamically unfavorable, we believe that the formation of Li_3_N is more likely attributed to an electrocatalytic process rather than the conventional Li-mediated strategy, which involves the initial generation of metallic Li followed by the thermochemical reduction of N_2_. This electrocatalytic process can be described as a continuous addition of electrons and Li ions to the adsorbed N_2_ molecules, as depicted in Fig. S8, with the free energy of each intermediate step labeled. Notably, except for the rate-determining step, the desorption of one Li_3_N molecule, the remainder of the reaction is exothermic. This observation highlights the remarkably low overpotential of Pt for the electroreduction of N_2_ to Li_3_N. It also explains why Pt can catalyze the reduction of N_2_ to Li_3_N, even at a potential of 0.5 V vs. Li/Li^+^, which is close to the theoretical N_2_/Li_3_N redox potential (0.53 V) [4]. These investigations underscore the significant catalytic activity of Pt in N_2_ reduction to Li_3_N. A comprehensive mechanistic study employing a suite of characterization techniques, including electrochemistry, spectroscopy and theoretical modeling, was performed and the results and analyses are provided in Supplementary Note 1 to further validate the catalytic role of Pt in the electrochemical reduction of N_2_ to Li_3_N.

### HOR-cascaded NH_3_ production

**Figure 3.**
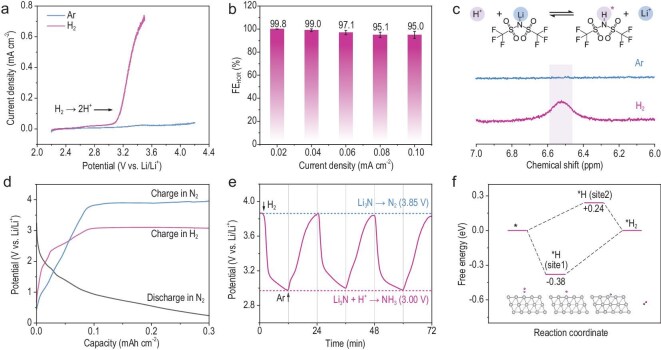
HOR-cascaded NH_3_ electrosynthesis. (a) LSV curves of Pt/Cu under an Ar and H_2_ atmosphere. (b) Corresponding FE of the HOR at different current densities. (c) ^1^H NMR spectra of the electrolyte after conducting HOR in the presence of LiTFSI. (d) GCD profiles of Pt/Cu recorded in N_2_ or H_2_ atmosphere. (e) Responses of charge potential by switching Ar to H_2_. (f) Comparison of N_2_ reduction and H_2_ oxidation activity at different sites (Pt atom and lattice vacancy) on Pt (111) and Pt (110) faces.

The electrocatalytic activity of Pt for HOR was investigated in a three-electrode system with Ag wire as the reference electrode and Pt as the working and counter electrode. In Fig. 3a, LSV curves of Pt under both Ar and H_2_ atmospheres are presented. When exposed to H_2_, the current density sharply increases at 3 V vs. Li/Li^+^, indicating the occurrence of HOR on the Pt electrode. To assess the efficiency of HOR, we applied a positive bias to the Pt electrode at various current densities. Figure S9 illustrates that the platform increases with higher current in the presence of H_2_, consistent with the LSV data. In contrast, under an Ar atmosphere and a low current of 0.04 mA cm^−2^, the platform reaches 4.2 V, likely corresponding to the electrolyte decomposition reaction. The Faradaic efficiency of HOR (*FE*_HOR_) was determined using GC, as shown in Fig. S10. The results indicate *FE*_HOR_ values of 99.8% ± 0.2%, 99.0% ± 1.0%, 97.1% ± 1.9%, 95.1% ± 2.3%, and 95.0% ± 3.1% at current densities of 0.02, 0.04, 0.06, 0.08 and 0.1 mA cm^−2^, respectively (Fig. 3b). To demonstrate that the protons generated from HOR can protonate Li_3_N, we conducted HOR experiments with an excess amount of Li_3_N powder added to the electrolyte. The product resulting from HOR is bis(trifluoromethane sulfonimide) (HTFSI), as confirmed by the ^1^H NMR spectrum of the resultant electrolyte (Fig. 3c and Fig. S11) and comparison with commercial HTFSI (Fig. S12). A slight chemical shift from 6.75 to 6.50 ppm may be attributed to the hydrogen bond between H^+^ and O_2_^−^ from HTFSI and TEGDME, respectively. To further validate the reaction between Li_3_N and HTFSI, we directly mixed commercial Li_3_N with HTFSI, and the resulting ^1^H NMR spectrum (Fig. S13) demonstrates the formation of NH_4_^+^. To further demonstrate the stability of Pt in TEGDME electrolyte during the HOR reaction, we tested the potentiostatic curves of Pt electrodes with varying surface areas. As shown in Fig. S14, the HOR current increases with the surface area of Pt and the current density remains stable over a prolonged period (500 min), indicating stable HOR performance in TEGDME. Scanning electron microscopy (SEM) images of the discharge products (Fig. S15) show that Li_3_N does not completely cover the underlying Pt, allowing exposed Pt sites to facilitate the HOR reaction. These findings confirm not only the excellent electrocatalytic HOR activity of Pt, but also the feasibility of NH_3_ production by cascading HOR to N_2_-to-Li_3_N electrocatalytic reduction.

The dual electrocatalytic activity of Pt in N_2_ reduction and H_2_ oxidation reactions allows an Li–N_2_/H_2_ battery to produce NH_3_ by coupling these reactions. The discharge and charge reactions can be summarized as follows. During discharge, N_2_ reacts with 6Li^+^ and 6e^−^, catalyzed by a Pt cathode, to form 2Li_3_N (N_2_ + 6Li^+^ + 6e^−^ → 2Li_3_N), accompanied by Li dissolving at the anode (Li → Li^+^ + e^−^). During charging, Li_3_N can be oxidized to N_2_ at the cathode, but if H_2_ is introduced into the system, HOR would occur before Li_3_N oxidation, as catalyzed by Pt. Therefore, the charging reaction at the cathode can be described as (H_2_ → 2H^+^ then Li_3_N + 3H^+^ → 3Li^+^ + NH_3_). The success of the Li–N_2_/H_2_ battery system in NH_3_ synthesis hinges on two fundamental prerequisites: first, the generated Li_3_N should not interfere with subsequent HOR reactions; and secondly, HOR reactions should take precedence over Li_3_N decomposition during charging. We intend to validate the ability of Pt to fulfill these conditions from both experimental and theoretical perspectives.

The battery was assembled using a Pt mesh cathode, Li foil anode and 1 M LiTFSI in TEGDME electrolyte (Fig. S16). The cathode shell has a hole for gas diffusion, and a piece of stainless-steel cloth covers the Pt to prevent electrolyte leakage. CV measurements (Fig. S17) indicate that the contribution of the stainless-steel cloth to catalytic N_2_ reduction is negligible. The battery is enclosed in a glass bottle with two tubes for gas flow, and the gaseous reactants (N_2_ and N_2_/H_2_ mixture) are contained in gas sampling bags. Gas circulation is facilitated by a pump. The GCD curves of the looped Li–N_2_/H_2_ battery, with a cut-off capacity of 0.3 mAh cm^−2^ at 0.04 mA cm^−2^, are shown in Fig. 3d. The battery was initially discharged in an N_2_ atmosphere to obtain Li_3_N at the Pt cathode. In the subsequent charging process, either N_2_ or H_2_ was supplied. With N_2_, the charging platform corresponding to Li_3_N oxidation was observed at 3.85 V, and no gas-phase NH_3_ was detected. This high voltage for Li_3_N oxidation is further supported by experiments where the Pt cathode was coated with a specific amount of commercial Li_3_N and charged in Ar, resulting in a similar charge platform at 3.8 V (Fig. S18). The charge platform decreased from 3.85 to 3.10 V when the battery was charged in the presence of H_2_, indicating the occurrence of HOR. Correspondingly, gaseous NH_3_ was detected after charging in H_2_, with a mass ratio of 1:4 between gaseous and dissolved NH_3_ (Fig. S19). To determine the charging voltage for the HOR and Li_3_N oxidation, the feed gas was switched between H_2_ and Ar during charging. Prior to the test, the battery was discharged in N_2_ to allow Li_3_N accumulation. The battery was then charged in Ar to reach a voltage of 3.85 V (Fig. 3e). When H_2_ was introduced into the system, the charge voltage immediately dropped to 3.15 V and then slowly decreased to 3.0 V, indicating the high sensitivity of the charging process to H_2_. Re-introducing Ar would gradually increase the charging voltage back to 3.85 V. This alternating variation of charging voltages under H_2_ and Ar atmospheres supports the definition of charging potential for HOR and Li_3_N oxidation. The voltage of HOR was also measured at different current densities. Figure S20 shows that the HOR voltage platform increased from 3.1 V at 0.04 mA cm^−2^ to 3.7 V at 0.6 mA cm^−2^, indicating that Li_3_N can be protonated at a high rate.

The stability of Li_3_N is crucial for its retention under high oxidative bias and subsequent protonation. We characterized the evolution of the discharge products during charging *in situ* using optical microscopy (Fig. S21). Minor structural changes were observed as the voltage increased from 1 to 3 V. Most products decomposed at 3.5 V, with full decomposition near 4 V, indicating a main oxidative decomposition potential around 3.5 V, agreeing well with the CV result in Fig. 2b. After charging the battery to 3 V, we scraped off the products for cryogenic transmission electron microscopy (cryo-TEM) characterization (Fig. S22). The products exhibited a core-shell structure with different contrasts: the shell corresponded to lithium oxides, and the core to Li_3_N, suggesting that the products were mainly Li_3_N coated with lithium oxides (Fig. S23). Further characterization using X-ray photoelectron spectroscopy (XPS) sputtering techniques showed a decrease in Li–O bonds and an increase in Li–N bonds with sputtering depth (Fig. S24), indicating that the outer layer is lithium oxides, the middle layer is lithium nitrides, and the inner layer is another layer of lithium oxides. This suggests that at a charging voltage of 3 V, the products on the Pt cathode are primarily lithium nitride coated with lithium oxides. The complex battery environment may contribute to the formation of this passivation layer, which exhibits electrochemical stability. It has been reported that Li_3_N nanoparticles, unless ground, lack electrochemical activity due to surface contamination by lithium oxides and carbonates, leading to gradual decomposition within a potential window from 1 V to approximately 3.5 V [17]. Additionally, commercially purchased Li_3_N has a decomposition potential around 3.8 V [23], as also proved by the result in Fig. S18. With special surface passivation treatments, the decomposition potential of Li_3_N could be even higher than 4 V [24]. This suggests that the degree of surface passivation influences the decomposition potential of Li_3_N, which is summarized in Fig. S25. These experimental results and existing reports strongly suggest that Li_3_N produced in an Li–N_2_/H_2_ battery system can survive at high potentials, allowing for protonation for NH_3_ formation, which guarantees NH_3_ production during cyclic testing (Fig. S26).

Density functional theory (DFT) calculations were conducted to provide theoretical insights into how the Pt cathode cascades the two reactions to generate NH_3_. We focused our investigation on two primary low-index crystal facets of Pt, namely (111) and (110), and compared the reactivity of different sites (Pt single atom and vacancy) on these facets toward electrocatalytic N_2_ reduction and H_2_ oxidation. A typical example showing the different free energies of the two sites for HOR is provided in Fig. S27, and the calculated rate-determining step energies are summarized in Fig. 3f. On the Pt(111) crystal facet, Pt atoms and their vacancies exhibited different activity toward electrocatalytic N_2_ reduction and H_2_ oxidation, suggesting that these two reactions might occur at distinct sites on the (111) facet. Simultaneously, on the (110) crystal facet, the vacancy sites displayed the highest activity for H_2_ oxidation (0.18 eV), further indicating that both reactions could potentially take place separately on different facets. We refer to this phenomenon as the job-sharing effect, which ensures that the two catalytic reactions can be effectively cascaded without interfering with each other. This is further supported by the nearly identical current density and onset potential of Pt for HOR, whether pre-discharged in N_2_ or not (Fig. S28).

The relationship between protonation and self-oxidation of Li_3_N on Pt was also theoretically investigated. It should be noted that Li_3_N was used as a model to calculate the activation energy of each intermediate state during NH_3_ formation, while 2(Li_3_N) was used to calculate the energy associated with Li_3_N oxidation and N_2_ reformation [25,26]. Based on the differential charge density (Fig. S29), it was observed that the electrons of Li_3_N accumulate at the N atoms in the optimized structure after Li_3_N adsorption on Pt. However, the electron transfer between Li_3_N and Pt is not prominent, indicating an obstacle for Pt to carry out Li_3_N oxidation. The Gibbs free energy of 2(Li_3_N) decomposition to N_2_ and Li_3_N protonation to NH_3_ on the Pt (111) surface was obtained based on the most stable configuration (Fig. S30). For Li_3_N protonation to NH_3_, the electrochemical steps were simulated by simultaneous Li_3_N de-lithiation, hydrogenation and Li reduction (Fig. S31a), following the overall reaction equation: Li_3_N (s) + 3H^+^ + 3e^−^ → 3Li (s) + NH_3_ (g), ∆*G* = 118.95 kJ mol^−1^, which is the typical reaction for battery charging. The highest Δ*G* of +1.34 eV is required in the final step from *LiNH_2_ to *NH_3_. In the case of Li_3_N oxidation to N_2_, the simulated reaction steps proceed from *Li_6_N_2_ (Li_6_N_2_@Pt) to *N_2_, bridged by a series of de-lithiation processes (Fig. S31b). Most intermediate steps are exothermic, except for the last two steps, *Li_2_N_2_ → *LiN_2_ → *N_2_, which require a maximum Δ*G* of +1.74 eV. This indicates that a substantial overpotential is necessary to drive the Li_3_N oxidation reaction. The Δ*G* value is higher than that of the *LiNH_2_ → *NH_3_ transition, suggesting that Li_3_N protonation is thermodynamically more favorable than oxidation. Therefore, NH_3_ is the predominant product during low potential charging in a H_2_ atmosphere. As for the possible reaction Li_3_N + 1.5H_2_ → 3Li + NH_3_; *E*^0^ = −0.391 V vs. SHE, our findings indicate no observable NH_3_ production after charging to 2.9 V (Fig. S32). This suggests that this reaction may not have occurred or does not significantly contribute to ammonia formation under our experimental conditions. In conclusion, the experimental analyses provide evidence for the feasibility of Pt as an electrocatalyst for N_2_ reduction to Li_3_N and H_2_ oxidation to protons, establishing the necessary prerequisites for the cascade NH_3_ electrosynthesis in the looped Li–N_2_/H_2_ battery. The key aspects of the bifunctional catalytic electrode of Pt are summarized in Supplementary Note 2.

The impact of excess protons and unprotonated Li_3_N on battery cycling performance is crucial. In the reaction Li_3_N + 3H^+^ → NH_3_ + 3Li^+^ (*E*^0^ = 2.6 V), the voltage decreases as Li^+^ concentration rises and H^+^ decreases according to the Nernst equation, reaching equilibrium at *c*(Li^+^)/*c*(H^+^) = 10^43^. At this point, a minimal concentration of residual H^+^ remains unreacted. These residual H^+^, with minimal impact on charge consumption, are further suppressed by concentration polarization, ensuring effective protonation of Li_3_N. During Li_3_N protonation, H^+^ depletion and Li^+^ release create a concentration gradient across the SEI layer: H^+^ increases from electrode to electrolyte, while Li^+^ decreases. Under pressurized conditions, N_2_ diffusion accelerates, promoting Li_3_N formation, while low H^+^ concentrations suppress H_2_ evolution. These factors collectively enable stable, efficient long-term battery cycling.

### Metrics of NH_3_ synthesis

**Figure 4.**
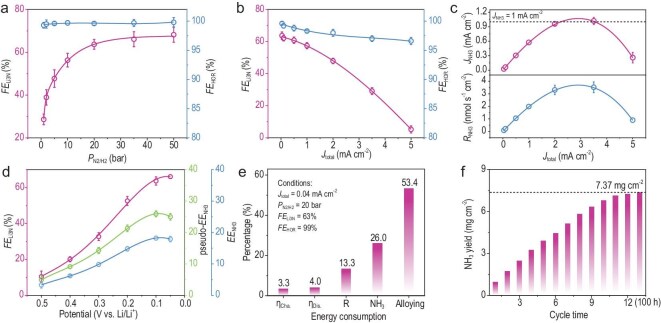
NH_3_ synthesis performances with Pt/Cu catalytic cathode. (a and b) *FE*_Li3N_ and *FE*_HOR_ at (a) different N_2_ pressures and (b) applied currents. (c) NH_3_ partial current densities and yield rates at different applied currents. (d) *FE*_Li3N_ at different discharge potentials and the corresponding pseudo-*EE*_NH3_ (without considering the energy of H_2_ supply) and *EE*_NH3_ (considering the energy of H_2_). (e) Parameters and the corresponding ratio of energy consumption. (f) Battery cycling experiment for long-term NH_3_ production.

Based on the aforementioned findings, we aimed to translate the fundamental reactions, coupled with electrode design, into high-performance Li–N_2_/H_2_ batteries for comparison with state-of-the-art electrochemical NH_3_ synthesis strategies, such as LiNR and aqueous NRR. To achieve this, we deposited a layer of Pt on porous Cu foam through electrochemical deposition, resulting in a Pt/Cu electrode with a mass loading of approximately 20 mg cm^−2^, which served as our working electrode (Fig. S33). Cu was chosen for its significantly lower catalytic activity for N_2_ reduction and H_2_ oxidation compared to Pt (as demonstrated in Fig. 2a and Fig. S34), primarily serving as a current collector. Its well-developed porous morphology could facilitate mass transfer (Fig. S35). Using Pt/Cu as the catalytic cathode, we first examined the influence of different gas pressures on *FE*_Li3N_ and *FE*_HOR_. A mixture of N_2_/H_2_ (v:v = 1:1) was used as the reactant. As shown in Fig. 4a, *FE*_Li3N_ significantly increased with increasing pressure (*P*_N2/H2_), reaching 63.7% ± 2.3% at a *P*_N2/H2_ of 20 bar. Further increasing *P*_N2/H2_ to 50 bar resulted in only a slight increase in *FE*_Li3N_ of 5%. In contrast, *FE*_HOR_ exhibited minimal variation with changes in *P*_N2/H2_, mainly due to inherent good HOR activity of Pt. The partial pressure of H_2_ would have an effect on the reaction efficiency (Fig. S36). The magnitude of applied current is also a critical parameter that is closely related to the FE and NH_3_ production rate. In general, increasing the current can enhance NH_3_ production rates but may compromise selectivity and energy efficiency. This trade-off is illustrated in Fig. 4b and c. Pt/Cu maintained approximately 60% *FE*_Li3N_ at 1 mA cm^−2^ (20 bar), but dropped below 50% as the current was gradually increased to 2 mA cm^−2^, further decreasing to around 5% at 5 mA cm^−2^ (Fig. 4b). During this process, the *J*_NH3_ initially increased and then decreased with the applied current density (Fig. 4c), reaching its peak at 1 mA cm^−2^ (at a *J*_total_ of 3.5 mA cm^−2^), corresponding to an NH_3_ production rate (*R*_NH3_) of 3.52 nmol s^−1^ cm^−2^ (0.12 mol h^−1^ m^−2^). The reaction between N_2_ and the Li anode for the formation of Li_3_N could be one of the sources for NH_3_ production as soon as the HOR is initiated. To address this issue, we have isolated the lithium anode from N_2_ gas using an H-type reactor for an Li–N_2_/H_2_ battery (Fig. S37). The results showed that there is a significant amount of NH_3_ produced in the N_2_/H_2_-saturated catholyte chamber, while that in the Ar-saturated anolyte chamber contributed only about 1% to the total NH_3_ production. This demonstrates that the electrochemical NH_3_ synthesis was dominated by Pt electrocatalysis, rather than chemical reaction between Li^0^, N_2_ and H^+^.

Energy efficiency has become a crucial parameter in assessing the economic viability of electrochemical methods, and it is closely related to overpotentials and corresponding FE. In our study, the battery was discharged to different cut-off voltages (at 20 bar), and its *FE*_Li3N_ was quantified. It is found that *FE*_Li3N_ increased as the discharge voltage decreased, which indicates that increasing the overpotential appropriately contributes to higher *FE*_Li3N_, and therefore there would be a maximum value of energy efficiency (*EE*). Assuming an *FE*_HOR_ of 99% and a voltage platform of 3 V for the charging reaction, the pseudo-*EE* for NH_3_ synthesis can be calculated using Equation (1) [27]:


(1) \begin{eqnarray*}
1.17 \times F{E}_{{\mathrm{L}}{{\mathrm{i}}}_3{\mathrm{N}}}/( {3 - {E}_{{\mathrm{dis}}.}} ) \,\times {\mathrm{\ }}100{\mathrm{\% }},
\end{eqnarray*}

where 1.17 represents the minimum voltage required to synthesize ammonia from water and nitrogen in an ideal electrochemical cell (see Supplementary methods). It is important to clarify that the ∼3.1 V charging potential we report is the full cell voltage, which encompasses the overpotentials for both HOR at the cathode and Li plating at the anode, plus ohmic losses. Consequently, our energy efficiency calculation, which is based on this experimentally measured total cell voltage, already fully accounts for the energy consumption of the lithium plating reaction. The maximum pseudo-*EE*_NH3_ was obtained at the discharge voltage of 0.1 V, with a value of 26.0%. When considering the energy of H_2_ supply, *EE*_NH3_ can be calculated using Equation (2) [11]:


(2) \begin{eqnarray*}
1.17{\mathrm{\ }} \times F{E}_{{\mathrm{L}}{{\mathrm{i}}}_3{\mathrm{N}}}/( {3 - {E}_{{\mathrm{dis}}.} + 1.23} ) \times {\mathrm{\ }}100{\mathrm{\% }},
\end{eqnarray*}

which results in a value of 17.9%. Based on pseudo-*EE*_NH3_, we calculated the energy consumption during the process of electrochemical catalytic NH_3_ synthesis [27]. The high selectivity and low overpotential of Pt in electrocatalytic N_2_ reduction and HOR contributed to a minimal fraction of energy lost to overpotentials in the charging and discharging reactions, accounting for only 7.3%. Due to the porosity and conductivity of Pt/Cu, the energy loss associated with internal resistance in the battery is only 13.3% (Fig. 4e and Fig. S38). Subtracting the 26% energy used for NH_3_ production, we attribute the remaining 53.4% of the energy to the alloying reaction between Pt/Cu and Li, which is common in lithium-ion batteries, especially at potentials close to 0 V vs. Li/Li^+^. We suggest that in the future, selecting suitable electrocatalytic materials to suppress alloying reactions and promote N_2_ reduction will be an important and ongoing research focus. Furthermore, long-term NH_3_ synthesis performance was tested, as shown in Fig. 4f. The total NH_3_ content in the exhaust gas and electrolyte was measured after different cycles. The NH_3_ increment remained relatively stable during the first 10 cycles. After the 13th cycle, which corresponds to 100 h of operation, the total NH_3_ production reached 7.37 mg cm^−2^, demonstrating the feasibility of the looped Li–N_2_/H_2_ battery for electrochemical NH_3_ synthesis.

A comparison of our method with the LiNR method is provided to address the potential advantages and trade-offs. Mechanistically, our two-step electrocatalytic process stabilizes the Li_3_N intermediate and enables its protonation, avoiding the significant energy loss and potential hydrogen evolution side reactions associated with lithium metal plating in the LiNR method. From a device perspective, our battery-based system utilizes heat released from the reaction (−32.9 kJ mol^−1^) to generate extra electricity, a benefit absent in the electrolytic setup of LiNR. These features underscore the superior energy efficiency and current utilization of the battery system. However, as with any approach, trade-offs exist. Due to the lack of highly efficient electrocatalytic materials and a low discharge voltage, our battery system currently lags behind the LiNR method in working current densities. Future work on improving catalyst performance and optimizing device structures could address the limitations and further enhance the competitiveness of the Li–N_2_/H_2_ battery system.

**Figure 5.**
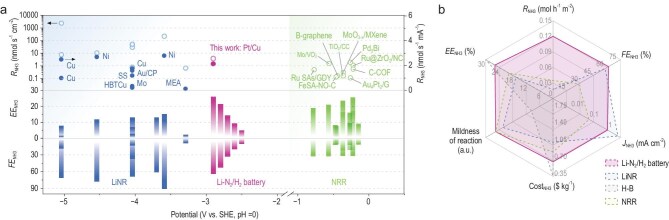
Comparison of performance. (a) *FE*_NH3_, *EE*_NH3_ and yield rates of our approach and previously reported methods are illustrated (the corresponding references are provided in Table S3), which are further differentiated based on the corresponding overpotentials. Two parameters are used here to determine the NH_3_ yield rate, including nmol s^−1^ cm^−2^ (labeled in hollow circles) and nmol s^−1^ mA^−1^ (labeled in solid circles). Note that nmol s^−1^ mA^−1^ is not used to determine the yield rates of aqueous NRR approaches due to their small *J*_NH3_ (∼0.1 mA cm^−2^) making this parameter meaningless. (b) Comparison of this work with state-of-the-art LiNR [11] and general NRR studies, including *FE*_NH3_, *EE*_NH3_, *R*_NH3_, *J*_NH3_, cost of NH_3_ production and mildness of reaction. The mildness was determined by parameters of temperature, pressure, overpotential and ease of material preparation. The electricity price is set at $0.03 (kWh)^−1^ [29].

In summary, the looped Li–N_2_/H_2_ battery achieved impressive results, including a maximum *FE*_NH3_ of 65.4% ± 1.8%, pseudo-*EE*_NH3_ of 26.0% ± 0.9%, *R*_NH3_ of 3.5 nmol s^−1^ cm^−2^, and a *J*_NH3_ of 1 mA cm^−2^. These results were achieved within a cell voltage range of 2.5–3 V under mild conditions. In comparison to representative previous reports on electrochemical NH_3_ synthesis, key performance indicators such as overpotential, *FE*_NH3_, *EE*_NH3_ and *R*_NH3_ were considered for comparison (Fig. 5a). In general, aqueous NRR technologies with lower overpotentials exhibit higher energy efficiencies but lower *FE*_NH3_ and *R*_NH3_. Conversely, LiNR technologies, which operate at higher overpotentials, achieve higher *FE*_NH3_ and *R*_NH3_ but lower *EE*. This suggests a clear trade-off between energy efficiency and NH_3_ yield rate with increasing overpotential. Since electricity cost significantly impacts the operational expenses of electrochemical NH_3_ synthesis, optimizing for higher energy efficiency (lower overpotential) and current utilization (defined as nmol s^−1^ mA^−1^) can substantially reduce production costs. Assuming an electricity cost of $0.03 (kWh)^−1^, we calculated the cost per kilogram of NH_3_ synthesized under different strategies (Fig. S39). Details of the calculations can be found in the Supplementary data. Considering only *R*_NH3_ and cell voltage (*U*_total_) as the main determinants, the looped Li–N_2_/H_2_ battery stands out as the most cost-effective option ($0.60–1.49 per kg_NH3_), followed by LiNR ($0.72–14.9 per kg_NH3_) and NRR ($0.78–0.90 per kg_NH3_). However, all three methods are more expensive than the existing H–B process ($0.3–0.5 per kg_NH3_), highlighting the urgent need to further reduce overpotentials and improve current conversion rates. It is important to note that other factors may also influence costs (such as H_2_ feedstock, balance-of-plant and chemical consumption, etc.; see Supplementary Note 3).

A comparison of overall performances of the looped Li–N_2_/H_2_ battery with one of the state-of-the-art LiNR research [11] and general NRR studies is given in Fig. 5b. It can be clearly seen that our method has the best overall performance, only slightly inferior to LiNR and the H–B method in terms of current density and cost, respectively. We believe that future research on Li–N_2_/H_2_ batteries should aim at improving the FE of N_2_ catalytic reduction and the NH_3_ partial current density at a low overpotential. The key is to develop bifunctional electrocatalytic electrodes with intrinsic low activation energies, accessible dual active sites and designed with optimized N_2_ and H_2_ mass transport. Designing devices with solid-state electrolytes (Fig. S40), three-electrode configuration (Fig. S41) or gas diffusion electrodes can also help to reach this goal. Exploring new metal–N_2_/H_2_ battery chemistries (e.g. Zn–N_2_/H_2_ or Al–N_2_/H_2_ battery systems) and new intermediates (e.g. LiN_3_, NaN_3_ etc.; Fig. S42) with even lower overpotentials (<2 V vs. SHE) holds promise for further advancements. This imperative for advanced electrode design brings into sharp focus the fundamental role of the cathode material itself. Our understanding of this role has evolved significantly. Previous work demonstrated that inert cathodes like stainless steel exhibit negligible activity for the direct electrocatalytic reduction of nitrogen [22], suggesting this pathway contributes little to the reaction efficiency. However, studies comparing different metals (Cu, Nb, Ni etc.) reveal that the cathode’s intrinsic properties are a key governing factor for performance [28]. This implies that on active metals like nickel, a coupled mechanism integrating both the traditional Li-mediated pathway and a direct electrocatalytic route may be responsible for the superior efficiency. Our findings provide a solid mechanistic foundation for metal–N_2_/H_2_ batteries for low cost and high rate of NH_3_ production at mild conditions.

## CONCLUSION

In conclusion, a looped Li–N_2_/H_2_ battery was introduced as a new electrocatalytic pathway for NH_3_ synthesis. In this approach, N_2_ is first electrocatalytically reduced to Li_3_N during discharge, and then it reacts with H^+^ generated by catalytic H_2_ oxidation during charging, producing NH_3_ in a discharge–charge loop. Distinguished from conventional NRR and LiNR methods, this approach temporally decouples N_2_ catalytic reduction from protonation while applying an oxidative bias during Li_3_N protonation, thereby avoiding the competing H_2_ evolution. By the rational design of the catalytic cathode, the overall NH_3_ productivity was boosted in terms of energy efficiency (*EE* = 26.0% ± 0.9%), selectivity (*FE* = 63.7% ± 2.3%), yield rate (*R*_NH3_ = 0.12 mol h^−1^ m^−2^) as well as current densities (*J*_NH3_ = 1 mA cm^−2^), which are comparable to the established approaches, demonstrating viable potential for industrialization.

## Supplementary Material

nwaf586_Supplemental_File

## Data Availability

All data supporting the findings of this study are available in the article and the Supplementary data. Data are also available from the authors upon request.
