# Supplementary material for: Intercoupled electrocatalytic ammonia synthesis via a looped Li–N2/H2 battery
Source: Natl Sci Rev. 2025 Dec 27;13(4):nwaf586. doi: 10.1093/nsr/nwaf586 (PMC12900419; doi:10.1093/nsr/nwaf586)
Supplement: nwaf586_Supplemental_File [file nwaf586_supplemental_file.pdf]

Supporting Information

Intercoupled electrocatalytic ammonia synthesis via a looped Li-N_2/H_2 battery

Zhendong Wang, Xiang Zhang, Zhiwei Xiao, Qian Feng, Jing Lin, Linlin Zhang, Yangyang Feng,* and Yaobing Wang**

Table-of-contents

Methods 2

Supplementary Figures 7

Supplementary Tables 49

Supplementary Note 51

Supplementary References 58

Methods

Electrode preparation

Platinum (Pt) mesh (50 mesh, working area 1 × 1 cm^2) was used directly as the working electrode. Prior to use, the Pt mesh was washed by DI, EtOH and acetone in an ultrasound bath. Then, it was polished in a HCl solution (pH = 1) at a reductive potential of -1.5 V vs. Ag/AgCl for 400 sec, washed by water and dried at 60 ℃ overnight. Pt/Cu was prepared as follows: A copper foam substrate (1 × 1 cm^2) was sequentially ultrasonically cleaned in deionized water and absolute ethanol for 20 min each, then dried under an Ar stream. Electrodeposition of Pt was performed using a conventional three-electrode system, with the polished copper foam as the working electrode, a Pt wire as the counter electrode, and an Ag/AgCl electrode (saturated KCl) as the reference electrode. The deposition was carried out in a 10 mM H_2PtCl_6 aqueous solution by applying a constant potential of –1.2 V for 5 min using a CHI660E electrochemical workstation. The resulting Cu–Pt electrode was rinsed thoroughly with deionized water and ethanol (2–3 times each) and dried in an Ar-filled glovebox before use. The mass loading of Pt was controlled at ~20 mg cm_{geo}^{-2}.

Pretreatment

All components utilized in the experimental setup were subjected to thorough drying procedures. Each component was dried overnight at 60 ℃ to eliminate any residual moisture. N_2 was purified to remove impurities. Initially, N_2 gas was cleaned by KOH solution to eliminate nitrogen oxides (NO_x) followed by passage through a sulfuric acid (H_2SO_4) solution to remove ammonia. Subsequently, the purified N_2 gas was further processed by THF to ensure complete removal of water. The resultant purified $^{15}N_2$ and $^{14}N_2$ gases were collected and then transferred to the glove box using gas sampling bag. The N-containing contaminants are summarized in Supplementary Tables S1 and S2. The moisture of the electrolyte was at a minimal 10 parts per million (ppm) through the use of Karl-Fischer titration.

Characterization

SEM and EDS were examined with a JEOL-6700F scanning electron microscope. X-ray diffraction crystallography was performed using a MiniFlex II diffractometer. ^1H NMR spectroscopy was performed on NMR spectrometer (Advance III, Bruker). The chemical composition was investigated by X-ray photoelectron spectroscopy (XPS, VG ESCALAB 250). The Faradaic efficiency of hydrogen oxidation reaction was analyzed by the gas chromatography (GC, 9790II, FULI).

Battery assembly and electrochemical investigation

Li-N_2/H_2 batteries are fabricated by sequentially stacking the cathode shell, platinum mesh, polypropylene separator, lithium foil and anode shell. 1 M LiTFSI TEGDME solution was used as the electrolyte. The cathode shell had a hole to allow gas diffusion. The batteries are sealed in a glass vial with two tubes running through a rubber cap and connected to a gas sampling bag containing the gaseous reactants. 1 M LiTFSI/TEGDME was used as the electrolyte. High pressure measurements are carried out using customized single chamber hermetically sealed stainless-steel vessels (up to 200 bar). LSV, CV and electrochemical impedance spectroscopy (EIS) tests were carried out using an electrochemical workstation (CHI 660E), while CP measurements were performed on a LAND test system. The N_2/H_2 mixed gas was purified by passing it through a KOH solution to remove NO_x impurities, followed by an H_2SO_4 solution to eliminate any NH_3. The purified gas mixture was collected in a gas-sampling bag and transferred into the glovebox for subsequent battery assembly.

NH_3 detection and quantification

NH_3 was detected by ^1H NMR and a colorimetric Nessler method and indophenol blue method. For ^1H NMR, the tested solution (pH = 4, HCl) was added into *d*-$CDCl_3$ with a ratio of 1:5 (v:v). For Nessler method, the test solution and 0.2 M potassium sodium tartrate were mixed with Nessler reagent in a ratio of 4:1:1 (v:v:v). After the reaction, the electrolyte was collected and mixed with 1 mL of 1 M HCl to convert all NH_3 into NH_4Cl. The solution was then dried using a rotary evaporator to obtain NH_4Cl crystals. The ammonia content was quantitatively determined by the indophenol blue method using UV–vis spectrophotometry.

Standard function for NH_4^+ concentration is shown by the aquation (3):

$$y = 0.15783 * x \text{ (R-square = 0.999)} \tag{3}$$

where y is the peak absorption and x is NH_4^+ concentration, in ppm. In the indophenol blue method, a sequence of solutions was mixed: 2 mL of an NH_3-containing solution, 2 mL of salicylic acid (5 wt.%), and 1 mol L^{-1} NaOH mixed solutions, followed by 1 mL of NaClO solution (0.05 mol L^{-1}), and finally, 0.2 mL of $C_5FeN_6Na_2O$ solution (1 wt.%). The prepared sample solution is then placed in a dark environment for a specific incubation period of 2 hours. Following incubation, the ammonia content in the solution is quantitatively determined using an ultraviolet spectrophotometer. The peak associated with ammonia is observed at a wavelength of 655 nm. A standard calibration curve is established with equation (4):

$$y=0.06778*x+0.014, \text{ (R-square=0.9994)} \tag{4}$$

Calculation of Faradaic efficiency

Faradaic efficiency for Li_3N production was acquired based on NH_3 production after hydrolysis according to the equation (5):

$$FE_{Li3N} = 3F \times m_{NH3} / (17 \times Q) \tag{5}$$

here, 3 represents the electron-transfer number for a single N atom, F denotes the Faraday constant, m_{NH3} is ascertained through colorimetric test, and Q signifies the overall charge. FE for hydrogen oxidation was acquired by gas chromatography. In our case, the signal at a retention time of ~0.5 min corresponds to the presence of H_2. Using different concentration of H_2 as standard samples, a standard curve in terms of H_2 ppm and peak area was obtained to quantify the H_2 concentration. In this work, standard function for H_2 concentration is shown by the aquation (6):

$$y = 164.6 \times x \text{ (R-square = 0.999)} \tag{6}$$

where y is the peak area and x is H_2 ppm. The FE_{HOR} is calculated by the formula (7):

$$FE_{HOR} = \Delta V_{H2} / 22.4 \text{ mol L}^{-1} \times 2 \times 96485 \text{ C mol}^{-1} / Q \tag{7}$$

where ΔV_{H2} is the consumption of H_2 during the charge process (measured by GC) and Q is the total charge passed during the charge process. Theoretically, the FE of NH_3 production is calculated by the formula (8):

$$FE_{NH3} = FE_{Li3N} \times FE_{HOR} \tag{8}$$

Practically, the FE can be calculated by the formula (9):

$$FE_{NH3} = Q_{out} / Q_{in} \times 100\ \% \tag{9}$$

where Q_{out} is the required electricity for NH_3 production, and the Q_{in} is the total capacity passed during the test.

Calculation of energy efficiency

The EE is defined as the ratio of the energy output (contained in the produced ammonia) to the total energy input (supplied by the potentiostat and, if applicable, the energy embedded in the hydrogen feed). The energy output is based on the Gibbs free energy change of the reaction in which ammonia is oxidized back to nitrogen and water:

$$NH_3 + 0.75O_2 \rightarrow 0.5N_2 + 1.5H_2O$$

The standard Gibbs free energy change for this reaction is:

$$\Delta G_R^0 = 339.244 \text{ kJ/mol}$$

The corresponding reversible cell potential is:

$$V_{cell}^0 = \frac{\Delta G_R^0}{zF} = \frac{339.244 \times 10^3}{3 \times 96485} \approx 1.17 \text{ V}$$

Here, $z = 3$ is the number of electrons transferred per NH_3 molecule, and F is Faraday's constant. Thus, the energy output for n_{NH_3} moles of ammonia is:

$$E_{out} = n_{NH_3} \cdot \Delta G_R^0 = 3F \cdot n_{NH_3} \cdot (1.17 \text{ V})$$

It represents the minimum voltage required to synthesize ammonia from water and nitrogen in an ideal electrochemical cell. This value is central to our energy efficiency calculation, as it quantifies the energy content of the ammonia produced. We have now included a more detailed derivation of the EE formula in the revised Methods section, referencing the Supplementary Materials for further details.

Estimation of NH_3 production cost

To simplify the calculation, we estimate the cost of ammonia production (\$ kg_{NH3}^{-1}) for different electrochemical ammonia synthesis strategies based mainly on two metrics, cell voltage (U_{total}) and ammonia production rate (R_{NH3}, nmol s^{-1} mA^{-1}). The following is the calculation procedure:

Moles (m) of NH_3 corresponding to 1 Ah is calculated as (equation 10):

$$m \text{ (mol)} = 36 * R_{NH3} / 100000 \quad (10)$$

Mass (M) of NH_3 corresponding to 1 wh is calculated as (equation 11):

$$M \text{ (g)} = m * 17 / U_{total} \quad (11)$$

Electricity Q(kwh) corresponding to producing 1 kg of NH_3 is calculated as (equation 12):

$$Q = 1 / M \quad (12)$$

Assuming an electricity price of \$0.03/kwh, the price corresponding to producing 1 kg of NH_3 is calculated as (equation 13):

$$\text{Cost (\$)} = 0.03 * Q = 0.03 / M = 0.49 * U_{total} / R_{NH3} \quad (13)$$

From this equation, it can be seen that the cost of electrochemical NH_3 synthesis is proportional to U_{total} and inversely proportional to R_{NH3}, i.e., efficient NH_3 production at a small overpotential will help to reduce the cost of NH_3 production. Additionally, factors such as the balance-of-plant, device design, chemical consumption etc. are also accounted to provide a realistic and holistic assessment of the economic feasibility of the proposed system (Supplementary Note 1). Here, several literatures on comprehensive techno-economic analyses are provided for further reference[1-4].

Isotope labeling experiments

Labeled nitrogen gas ($^{15}N_2$, 98 atom %) were used for isotope labeling experiments. $^{15}N_2$ gas was pretreated according to the previous report[5]. A $^{15}N_2$ supersaturated electrolyte was used for all relevant experiments.

Theoretical calculations

Vienna ab Initio Simulation package (VASP) with the projected augmented wave (PAW) potentials was used for the first principle calculations[6]. Perdew-Burke-Ernzerhof (PBE) generalized gradient approximation (GGA) was used for investigating the electron correlation and exchange interaction[7, 8]. The cutoff energy was set to 500 eV for the plane-wave basis. The energy and force threshold are 10^{4-} eV and 0.01 eV/Å, respectively, for the structure relaxation. The (2 × 2) supercell Pt 111 slab with 15Å vacuum space was full-relaxed. Considering the influences of van der Waals interactions, the DFT-D3 functional was used for the dispersion[9]. The Gibbs free energy of the reactions were calculated with the Nørskov method[10], in which the Gibbs free energy of $H^+ + e^-$ was replaced with that of one-half an H_2 molecule.

Supplementary figures

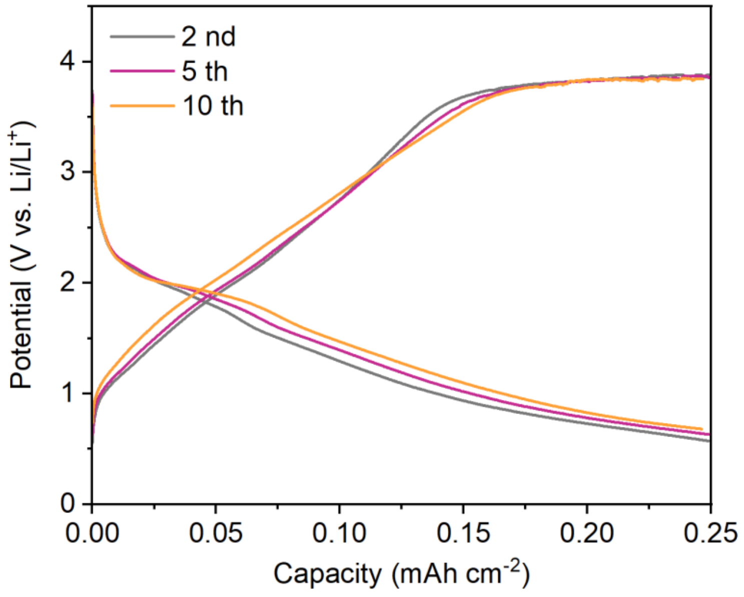

Fig. S1 Cycling stability of Li-N_2 battery.

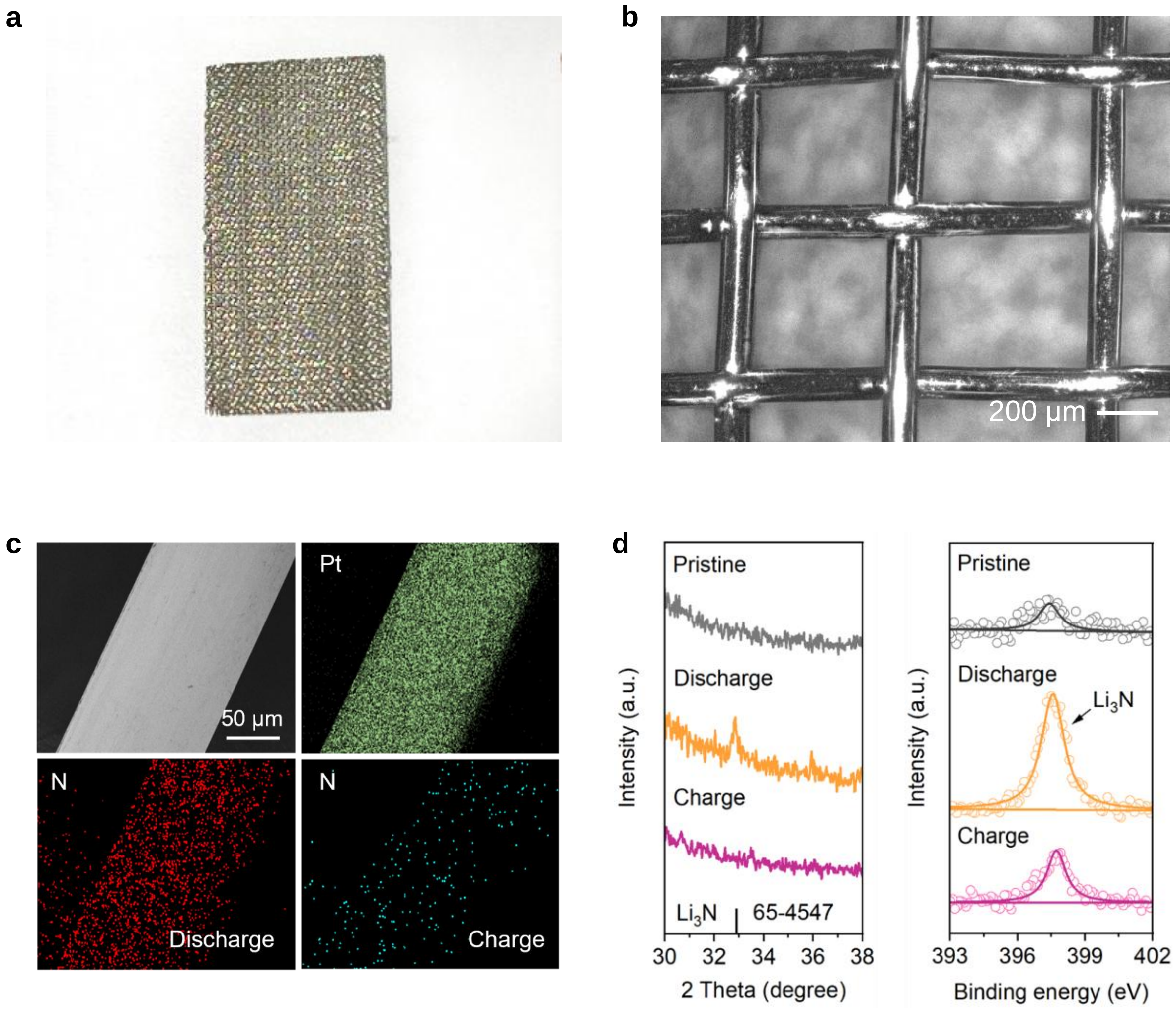

Fig. S2 a, A digital photo and **b**, optical microscope image of Pt mesh. **c**, SEM image and EDS mapping of the Pt cathode at full discharge and charge states. **d**, XRD patterns and XPS N1s spectra of the Pt cathode at full discharge and charge states.

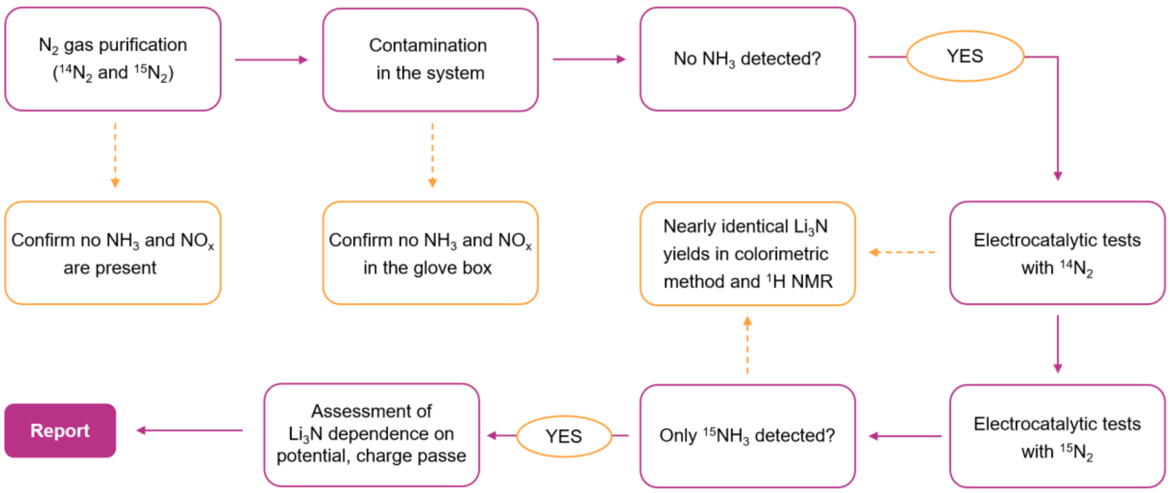

Fig. S3 The experimental protocol for reliable proof of the occurrence of the electrochemical nitrogen reduction to Li_3N reaction in this work.

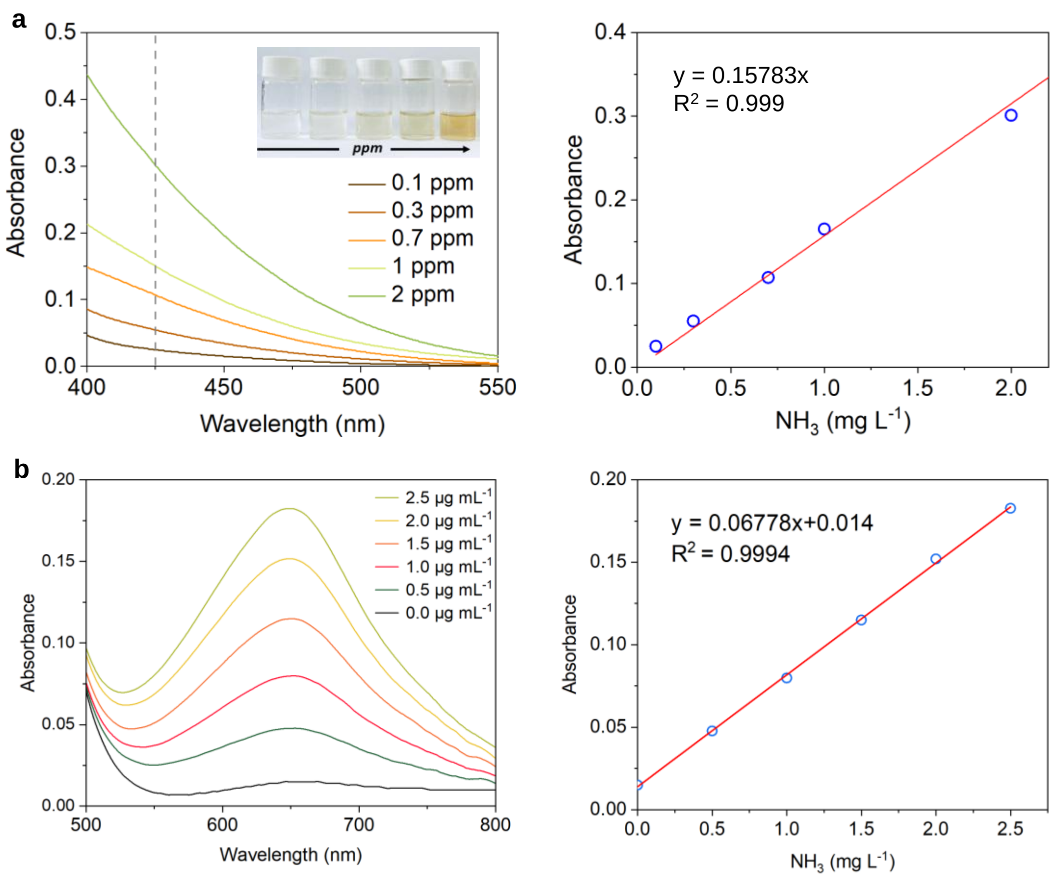

Fig. S4 a, Standard curves obtained by Nessler reagent. **b**, Standard curve for quantification of NH_4^+ using indophenol blue method.

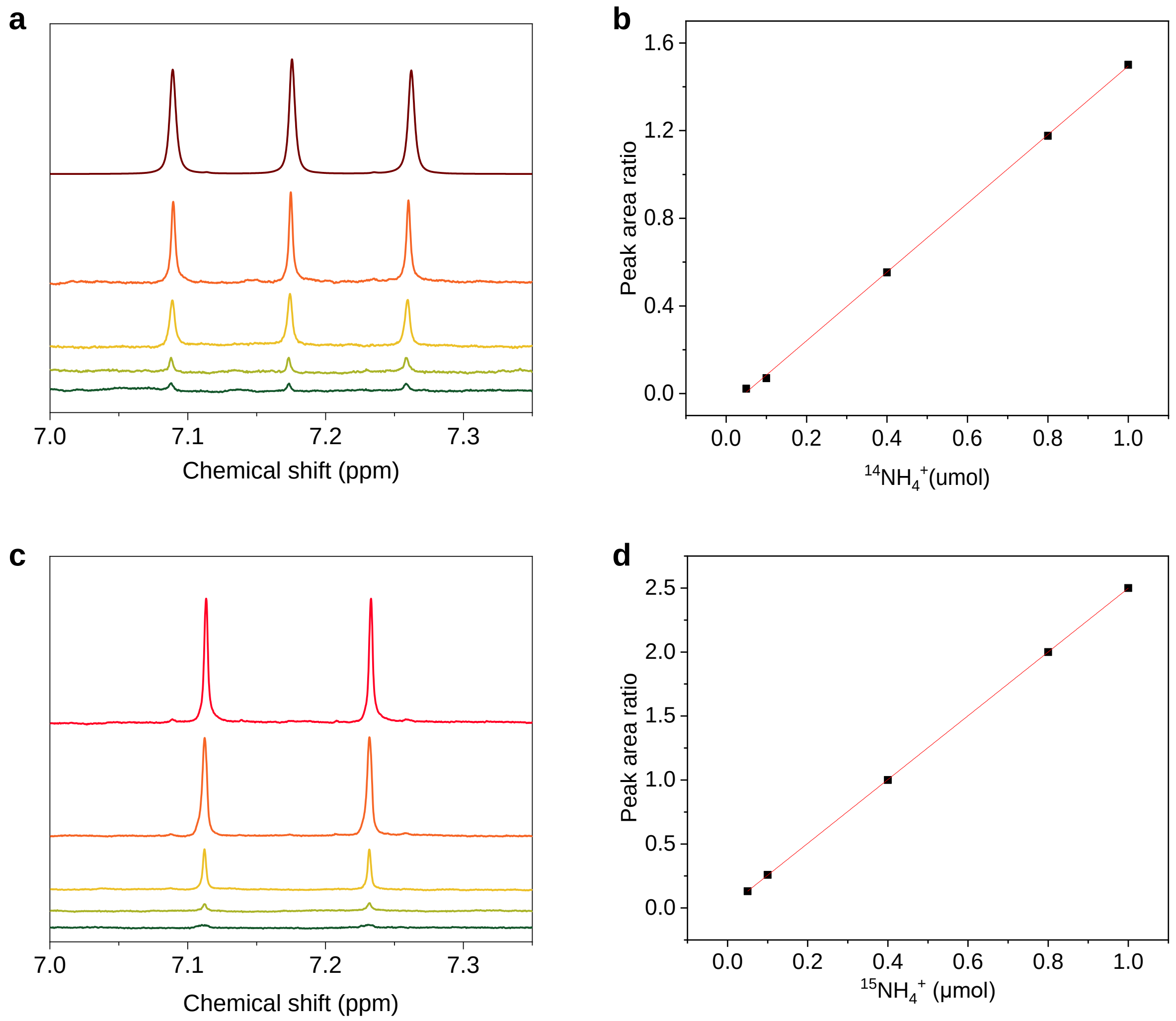

Fig. S5 Standard curve for quantification of NH_4^+ using ^{1}H NMR. a, ^{1}H NMR spectra of different $^{14}NH^{4+}$ concentrations. **b**, Integral area ratio ($^{14}NH^{4+}/C_4H_4O_4$) against $^{14}NH^{4+}$ concentration. **c**, ^{1}H NMR spectra of different $^{15}NH^{4+}$ concentrations. **d**, Integral area ratio ($^{15}NH^{4+}/C_4H_4O_4$) against $^{15}NH^{4+}$ concentration.

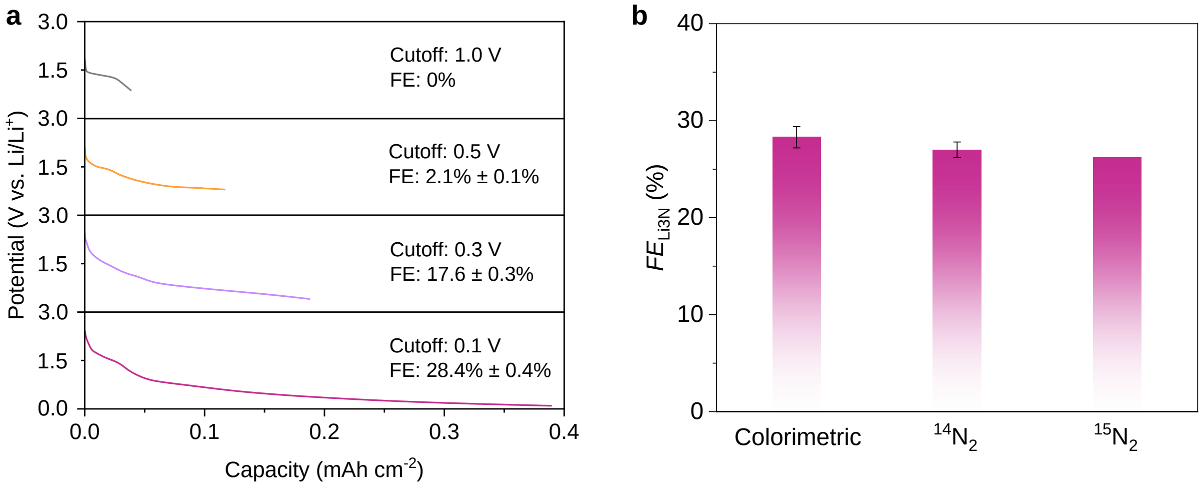

Fig. S6 **a**, Discharge profiles of Pt with different cut-off voltages and the corresponding Li_3N FE in a Li-N_2 battery. **b**, FE_{Li3N} determined by colorimetric methods and ^{1}H NMR. The error bars correspond to the standard deviations of measurements over three separately prepared samples under the same conditions.

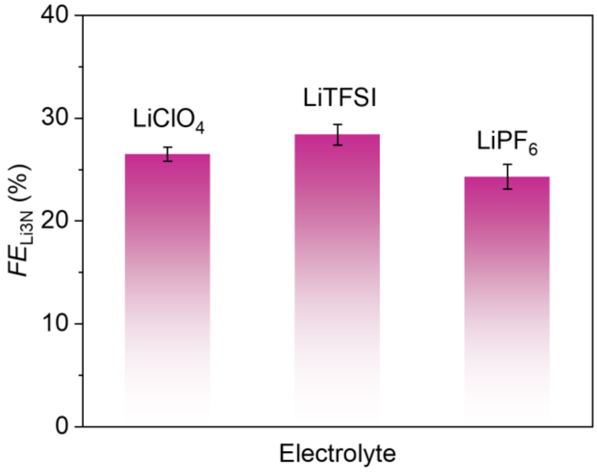

Fig. S7 *FE*$_{Li3N}$ values for Li-N_2 batteries with different electrolyte (LiTFSI, $LiClO_4$ and $LiPF_6$) discharging at atmospheric pressure and N_2 atmosphere.

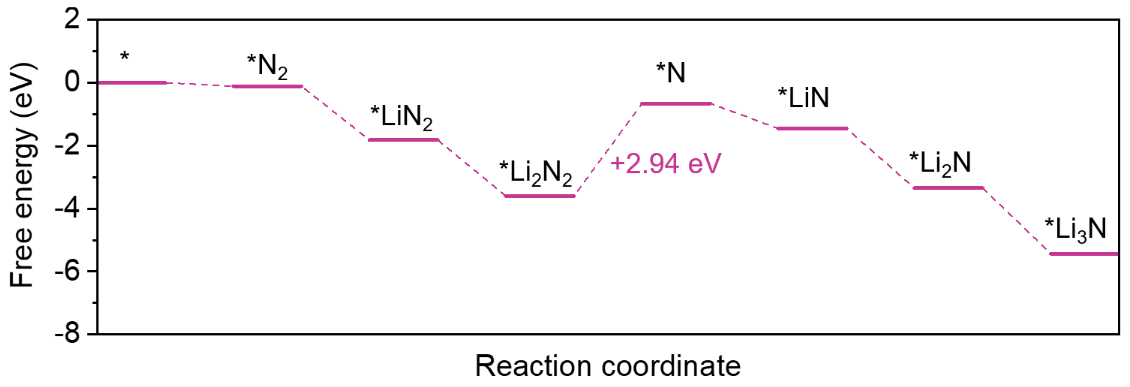

Fig. S8 Schematic of associative mechanism for Li_3N formation. **a**, Association mechanism with a distal pathway (Li on the distal N) of Pt. **b**, Schematic Gibbs free energy diagrams. Except for the rate-determining step, i.e., the transition reaction from $*Li_2N_2$ to *N by the addition of one Li^+ and e^- and the desorption of one Li_3N molecule, the rest of the reaction is exothermic.

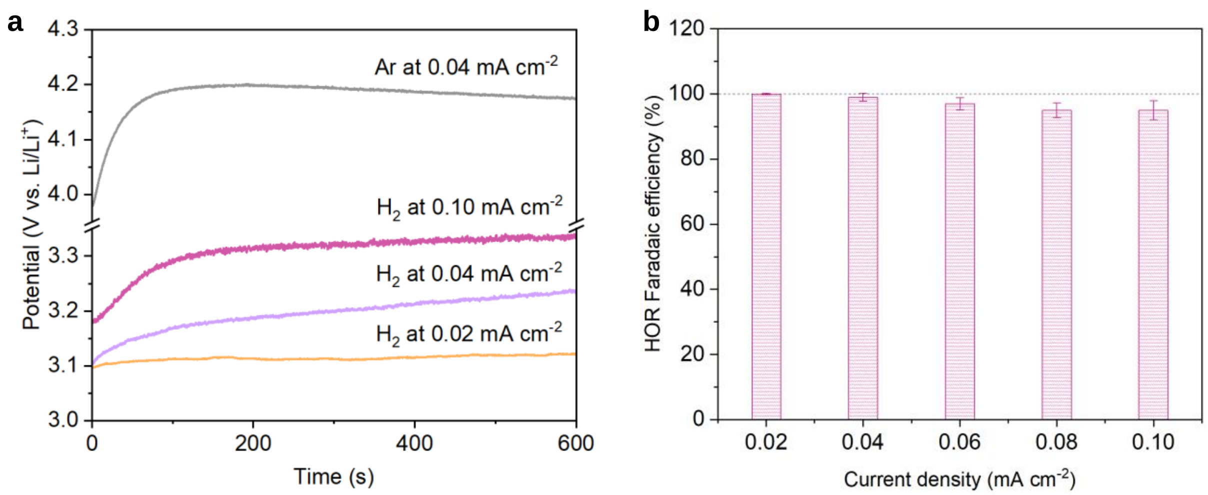

Fig. S9 a, Constant potential curves and **b**, corresponding FE of the HOR at different current densities.

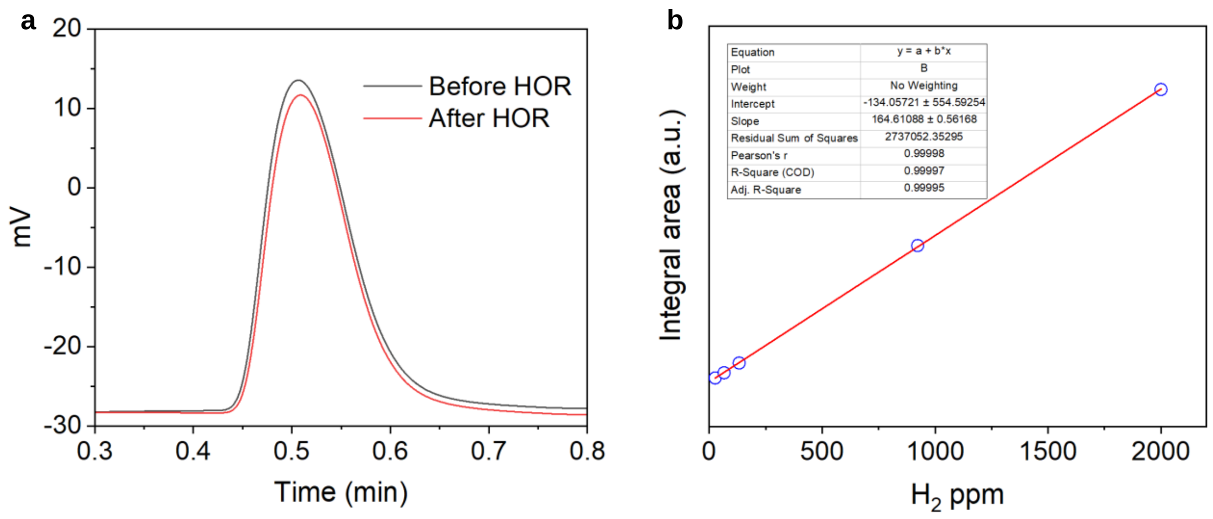

	Peak height (μV)	Peak area (μV*s)	H_2 (ppm)
Before HOR	42343.4609	252190.6250	1491.4642
After HOR	40065.5117	233531.2031	1381.1117

Fig. S10 a, Typical H_2 signal measured by a GC after charging the cell at 0.1 mA cm^{-2} over 1 h under a H_2 atmosphere. The theoretical H_2 consumption based on the total charge (Q) is 120 ppm, and the practical value is ~110 ppm (1491.4642 - 1381.1117 = ~110), accounting for a 92% FE of HOR. FE values under other current densities and pressure were calculated accordingly. **b**, Fitted standard curves in terms of H_2 concentration, in ppm, and integral peak area recorded by the gas chromatography.

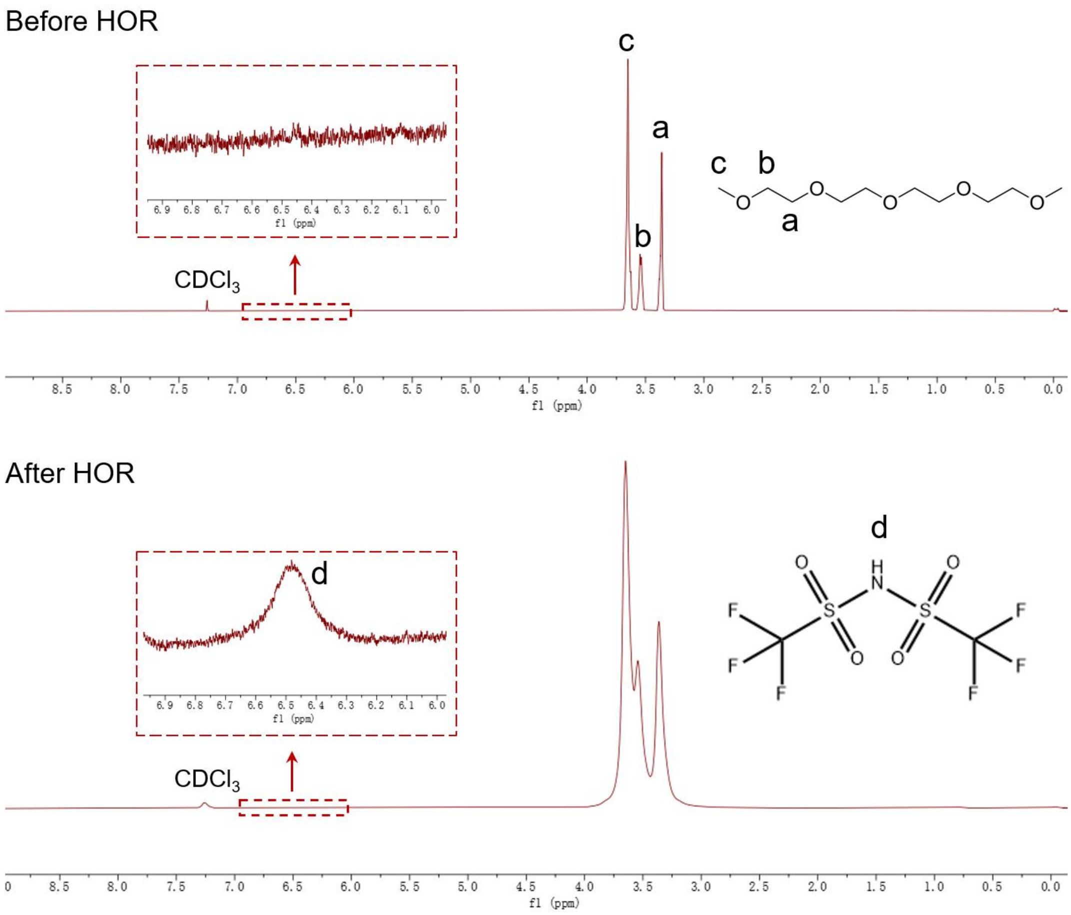

Fig. S11 ^{1}H NMR spectra of the electrolyte (1 M LiTFSI in TEGDME) before and after the HOR.

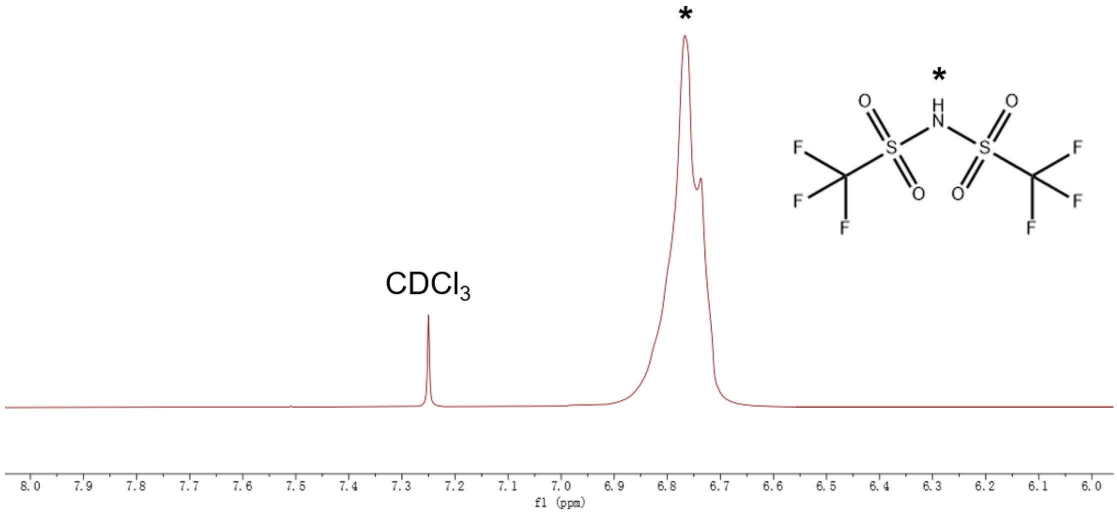

Fig. S12 Standard ^{1}H NMR for the commercial HTFSI.

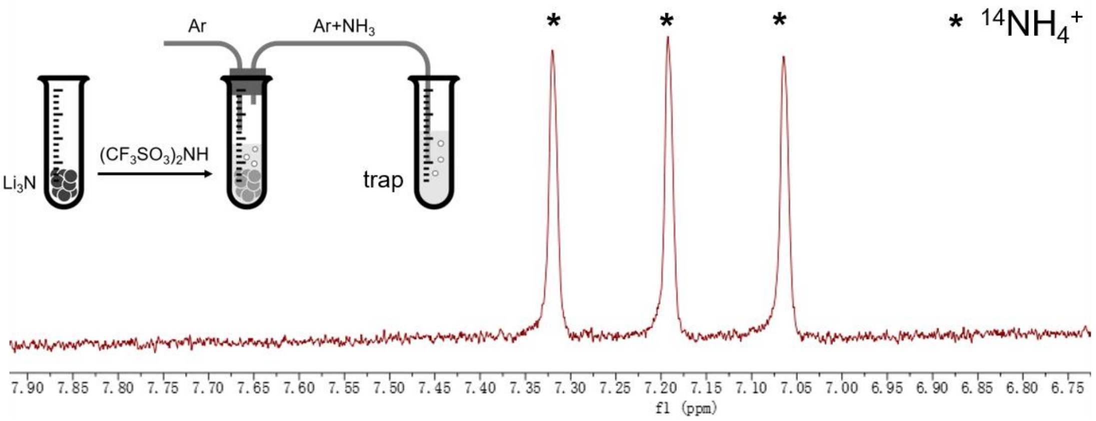

Fig. S13 Standard ^{1}H NMR spectrum obtained by mixing the commercial Li_3N with HTFSI.

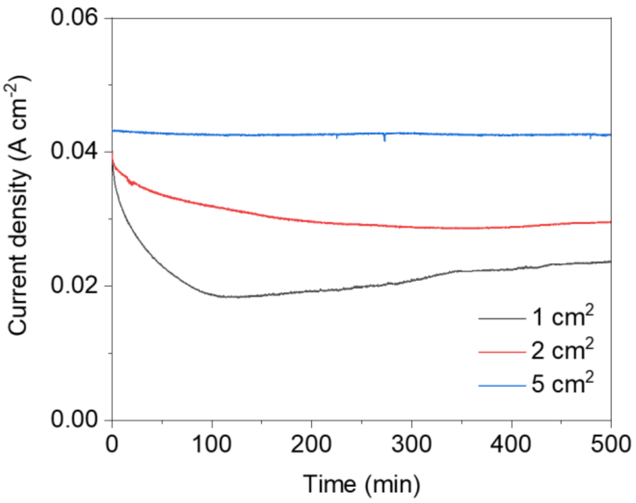

Fig. S14 Potentiostatic curves of Pt of varying surface areas for HOR in TEGDME electrolyte.

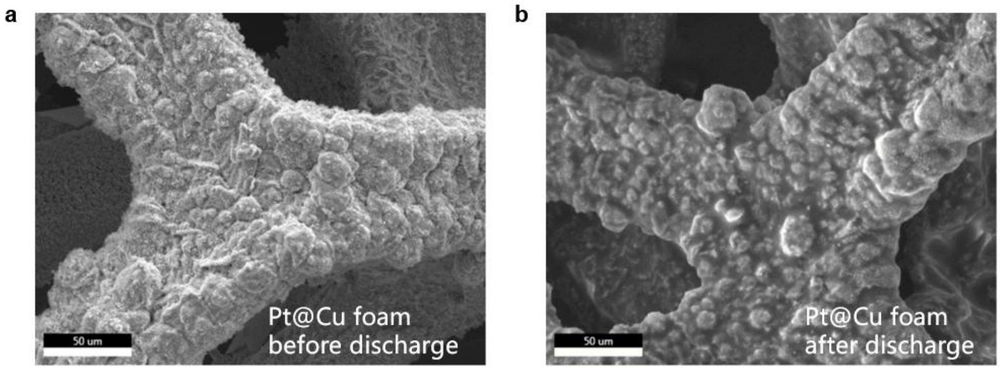

Fig. S15 Potential electrocatalytically active surface. a, SEM image of Pt@Cu foam cathode showing exposed Pt nanoparticle active sites before discharge. **b**, SEM image of Pt@Cu foam cathode with distinct film-like covering after discharge. There are still a large number of exposed Pt sites available for subsequent HOR reactions.

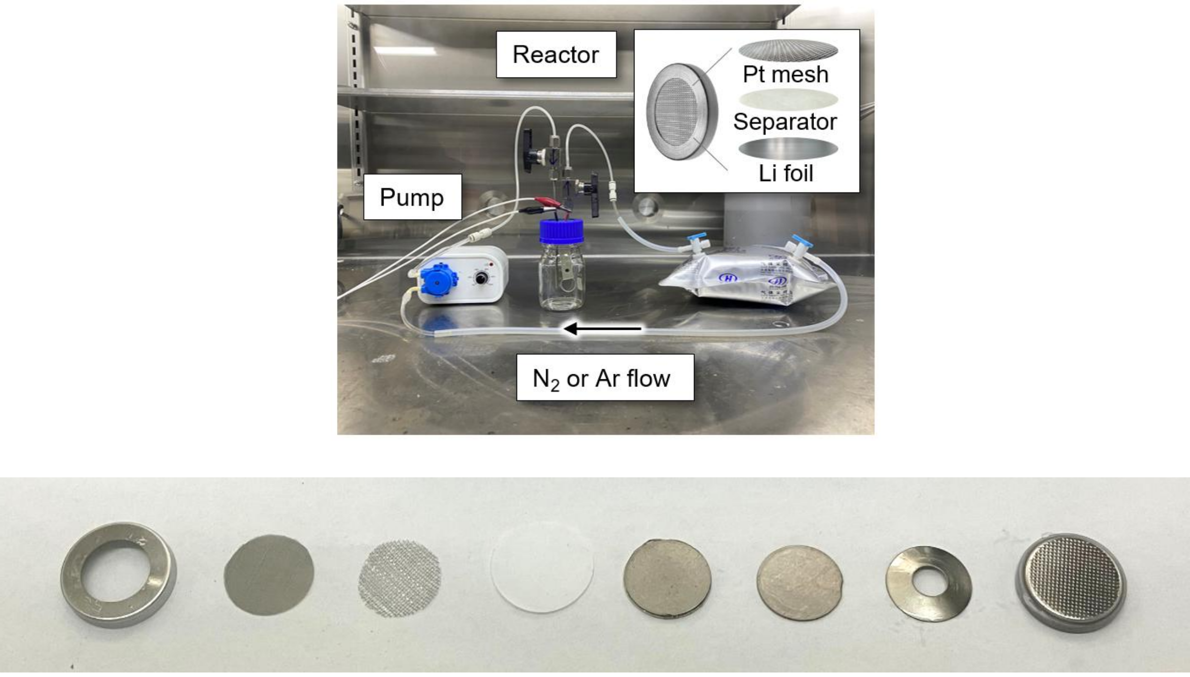

Fig. S16 Experimental setup and the assembly of the Li-N_2/H_2 battery in a glove box.

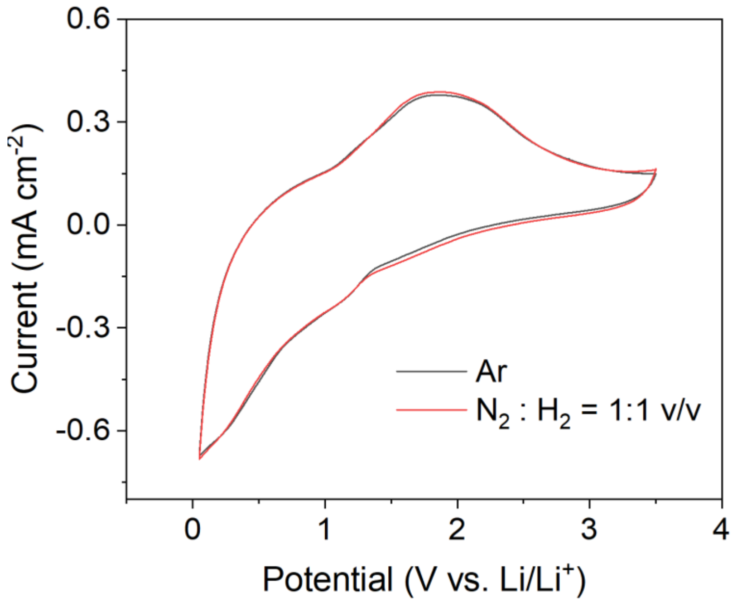

Fig. S17 CV curves of stainless-steel cloth under Ar and N_2/H_2 atmospheres.

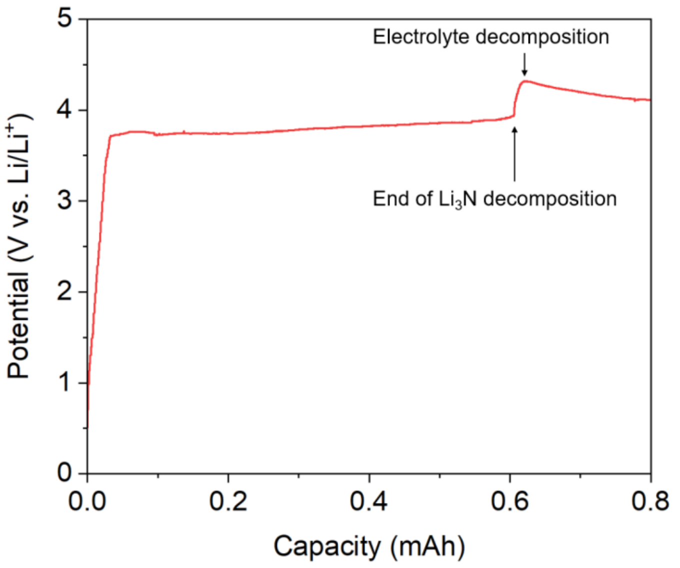

Fig. S18 Charge profile of Li-N_2 battery with specific amount of commercial Li_3N coating on Pt cathode.

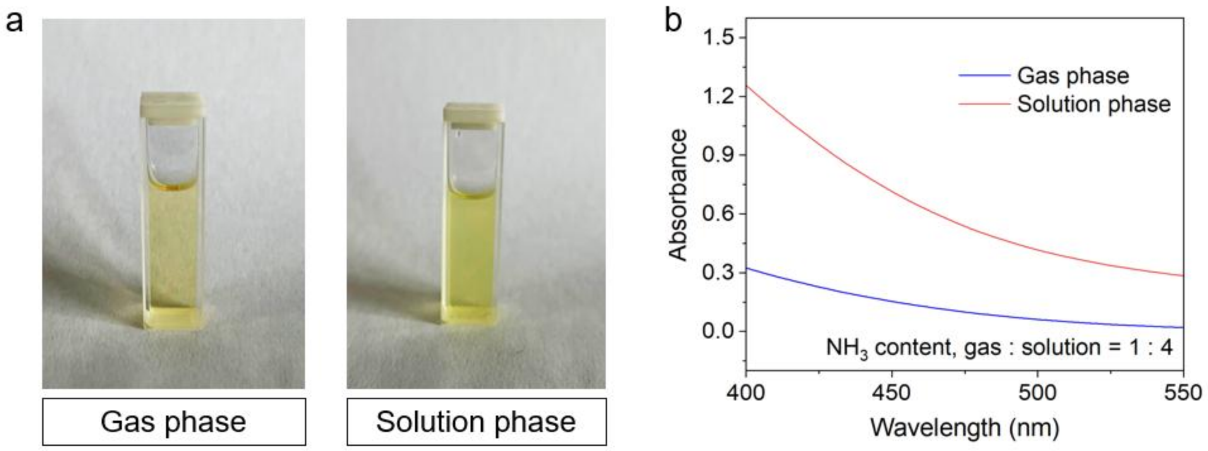

Fig. S19 a, Digital images of NH_3 detection in gas/solution phase. **b**, Corresponding UV-Vis spectra. NH_3 in solution phase is found in the electrolyte whereas gas phase NH_3 is trapped by water.

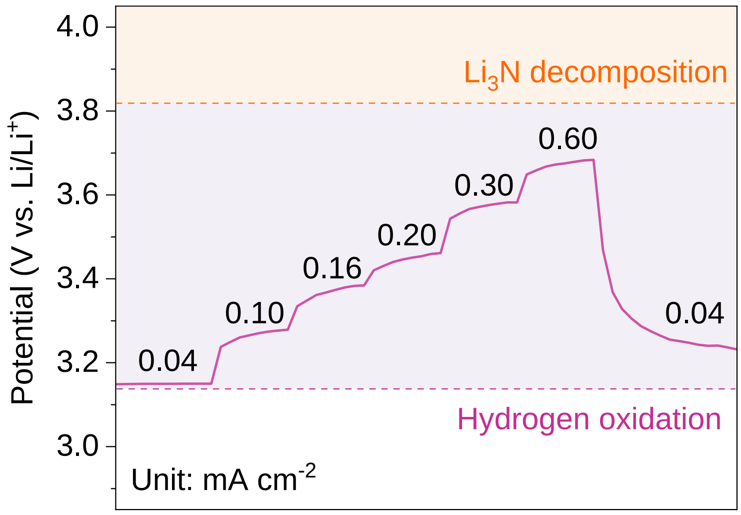

Fig. S20 HOR reaction potentials at different charge current densities.

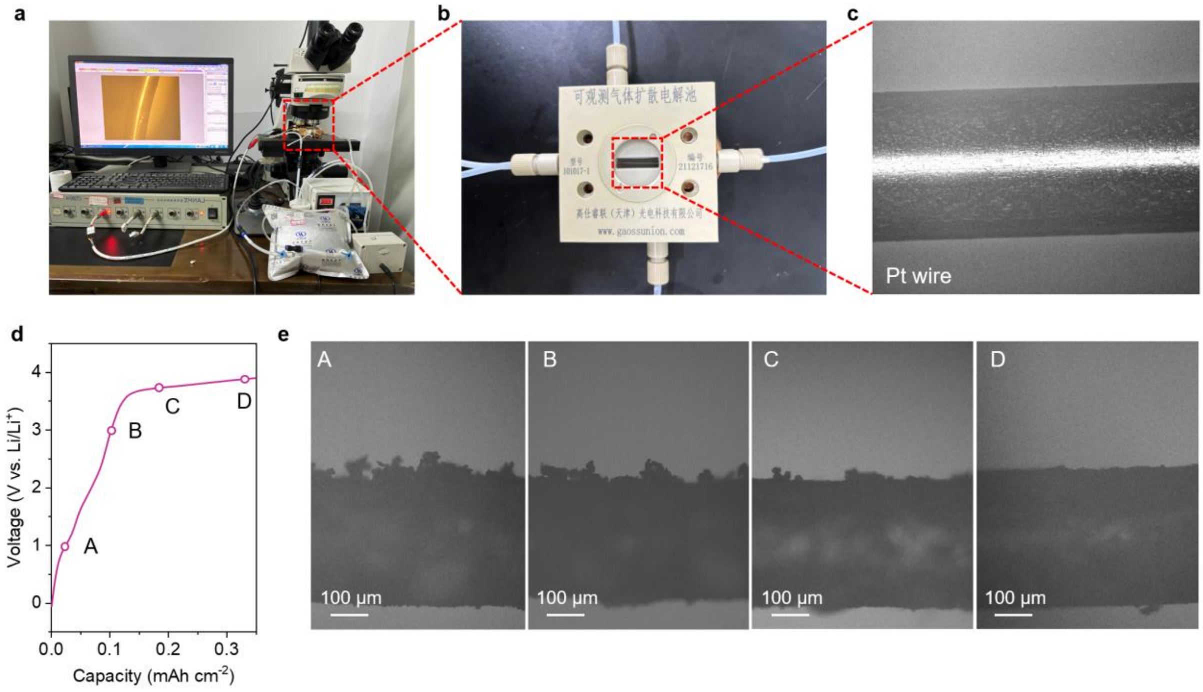

Fig. S21 Optical microscopy characterization of the discharge products during charging. **a**, Test and characterization setup. **b**, A Li-N_2 gas-flow battery with an observation chamber. **c**, The surface of Pt wire before discharge. **d**, Charging curve under N_2 atmosphere. **e**, Optical microscope images of the Pt wire surface products at various charging potentials.

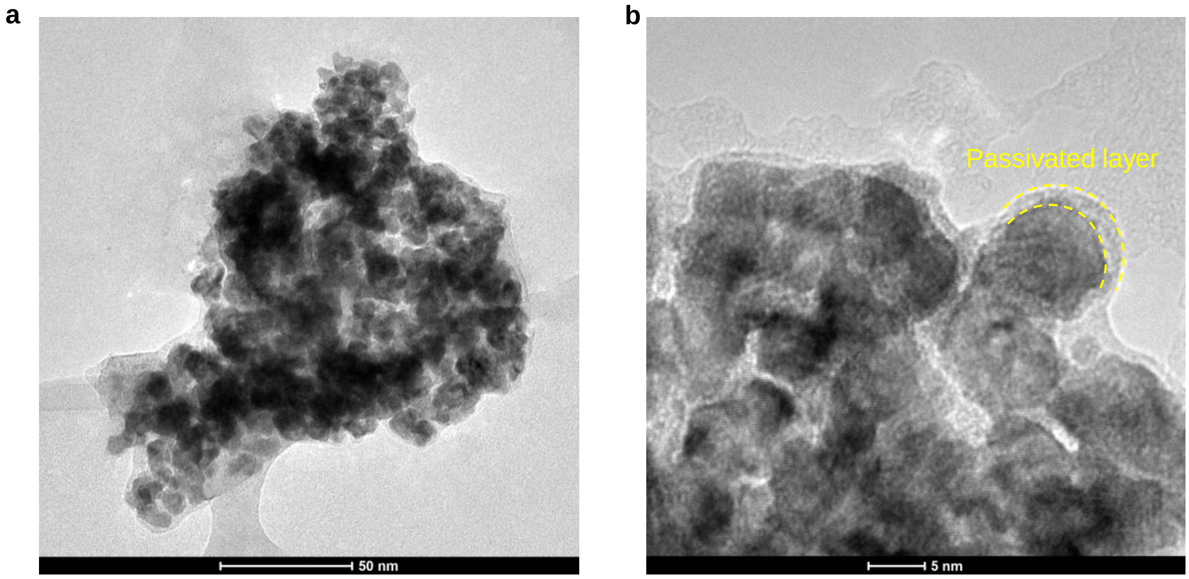

Fig. S22 Cryo-TEM characterization. a, Cryo-TEM image and **b**, High-resolution cryo-TEM image of the discharge products after charging to 3 V, which indicates a core-shell structure.

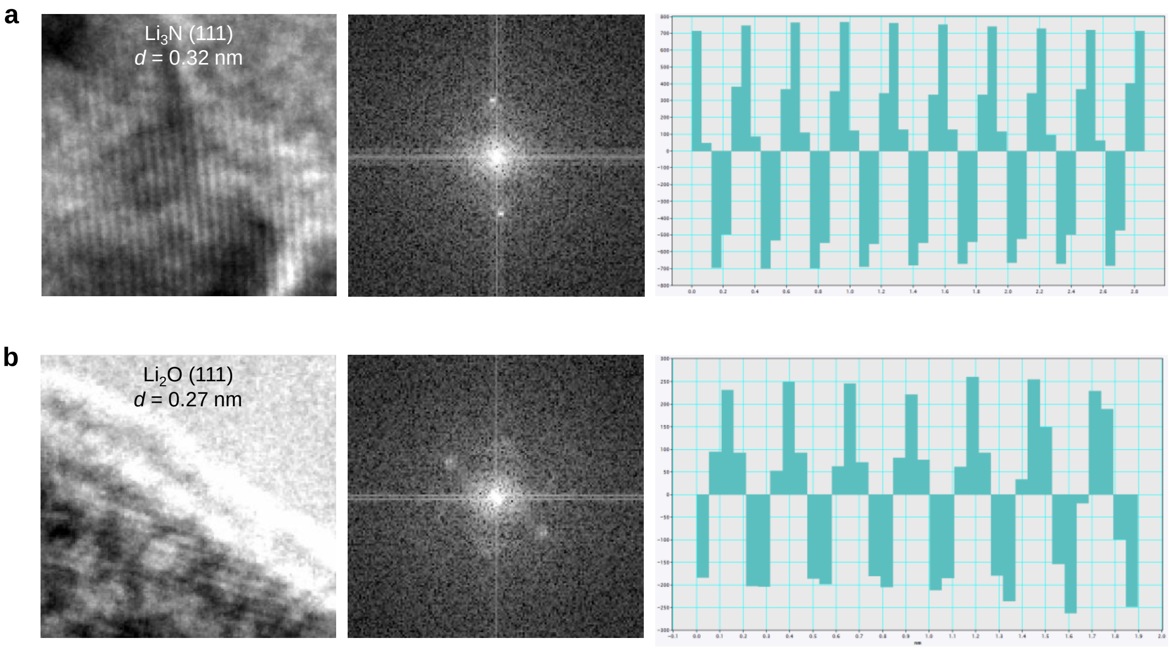

Fig. S23 FFT images and corresponding IFFT images, intensity profiles of **a**, the core and **b**, the shell of the products in the cryo-TEM images. The shell corresponds to lithium oxides, while the core corresponds to Li_3N. This indicates that the products are mainly Li_3N coated with lithium oxides.

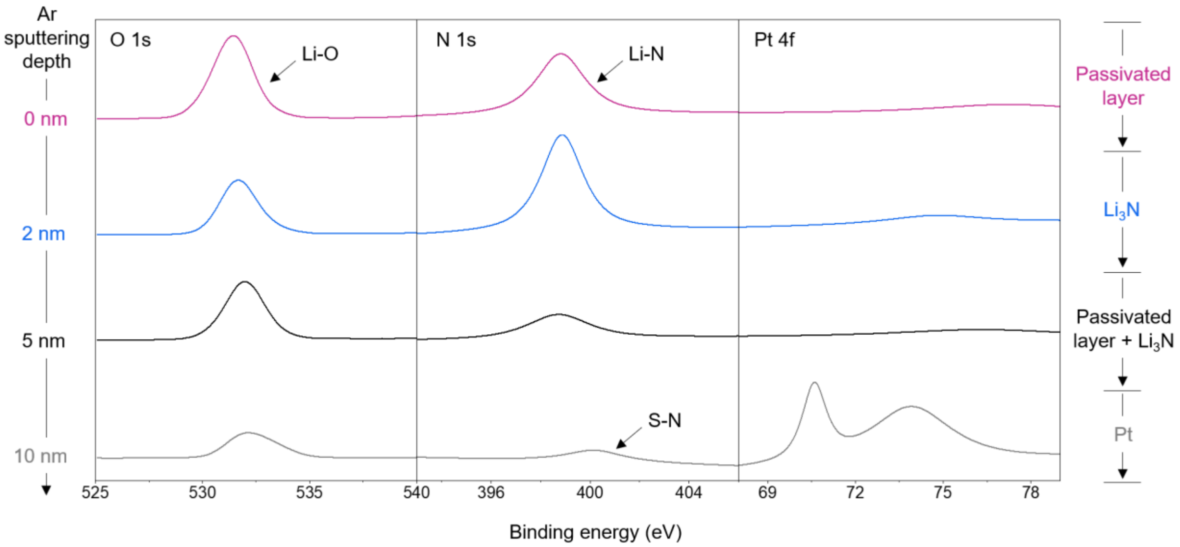

Fig. S24 XPS depth profiling spectra of the discharge products within 0-10 nm. At a sputtering depth of 0 nm, we observed a large number of Li-O bonds and a small amount of Li-N bonds. As the sputtering depth increased, the Li-O bond content gradually decreased, and the Li-N bond content increased until the Pt signal appeared, at which point the Li-O bond content increased again. This indicates that the outer layer of the electrode surface material is mainly lithium oxides, the middle layer is lithium nitrides, and the inner layer is another layer of lithium oxides.

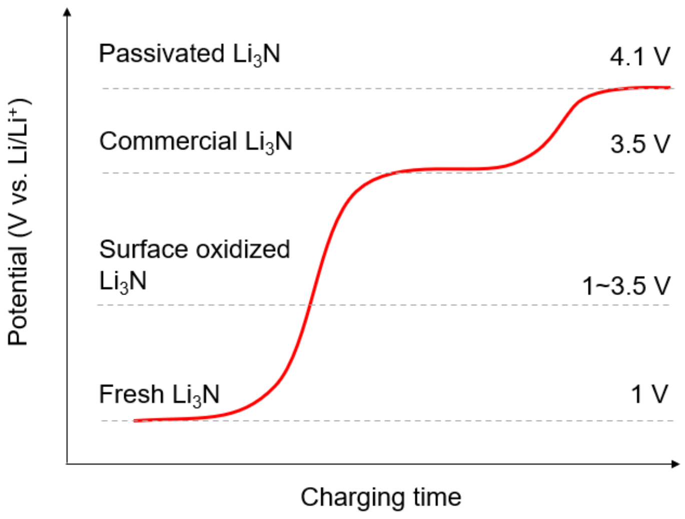

Fig. S25 Electrochemical oxidation potentials of Li_3N in different states summarized from literature reports and our data.

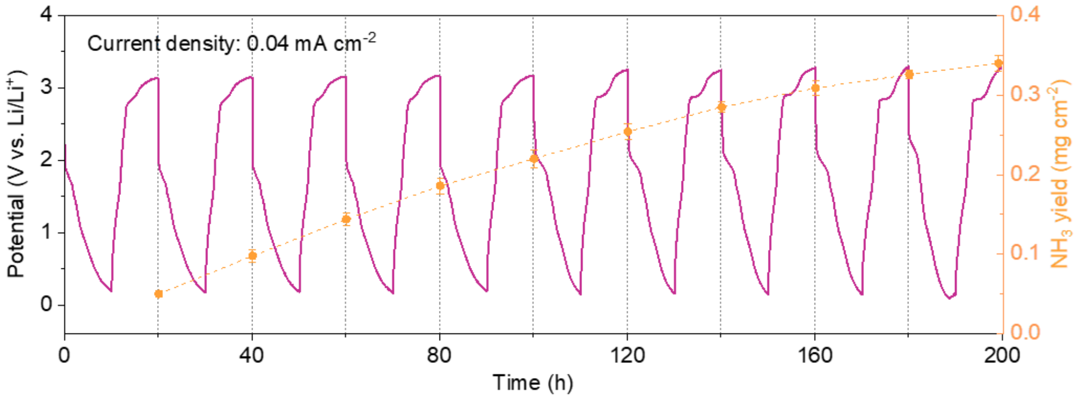

Fig. S26 Cycling stability of Li-N_2/H_2 battery equipped with Pt cathode for NH_3 electrosynthesis. The galvanostatic charge-discharge profiles of the Li-N_2/H_2 battery were recorded over 10 consecutive cycles under a N_2/H_2 atmosphere (1:1 v/v). The curves exhibit highly consistent discharge and charge plateaus throughout cycling, with minimal voltage polarization increase. This remarkable reproducibility in the electrochemical profiles directly demonstrates excellent cycling stability at the system level. Furthermore, this electrochemical stability aligns perfectly with the sustained NH_3 production performance previously reported. The system maintained an average NH_3 yield of 0.028 nmol cm^{-2} s^{-1} (0.34 mg cm^{-2}) over 200 hours of continuous operation, confirming the structural integrity of the electrode and the robustness of the overall reaction system.

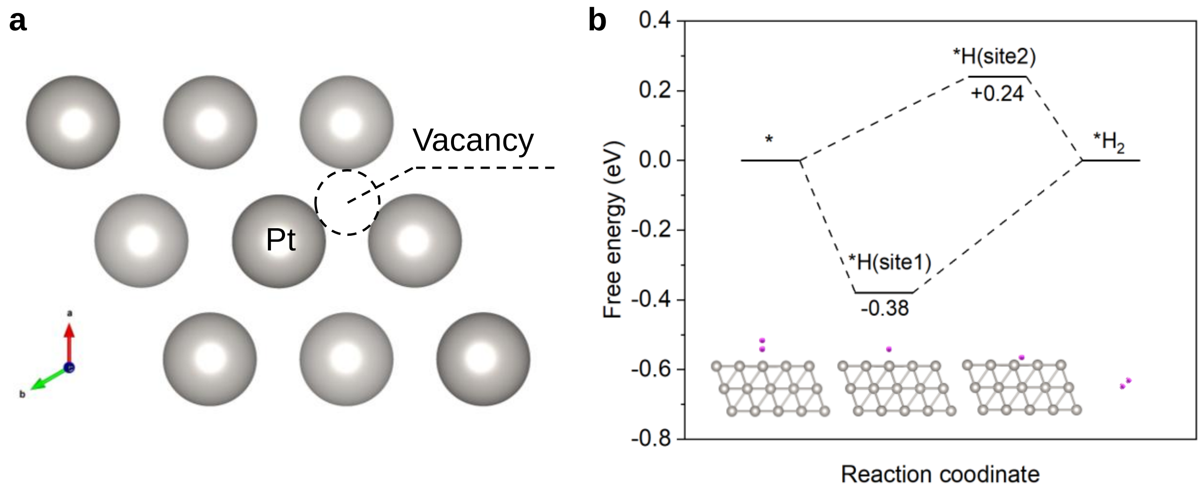

Fig. S27 **a**, Two different sites (Pt atom and its vacancy) on Pt(111) and (110) are considered as active sites for N_2 reduction and H_2 oxidation. **b**, Illustration of different catalytic activities of the two active site toward H_2 oxidation on Pt(111).

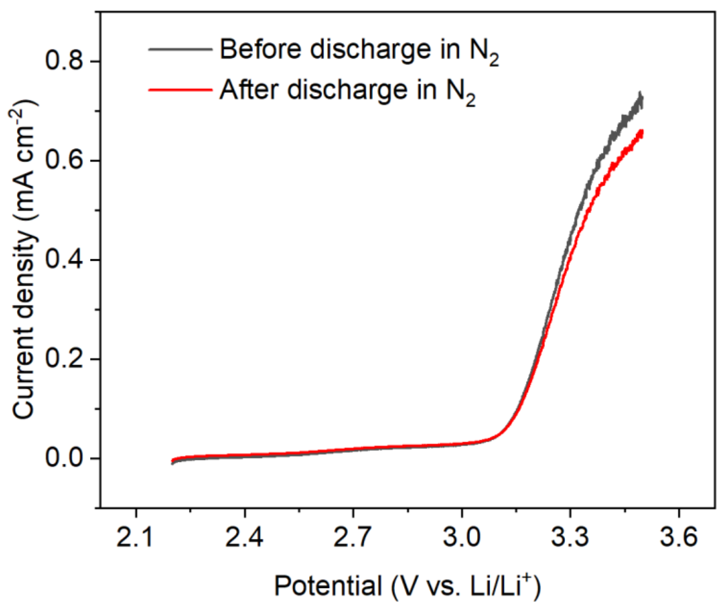

Fig. S28 LSV curves for HOR at Pt with/without pre-discharging in N_2.

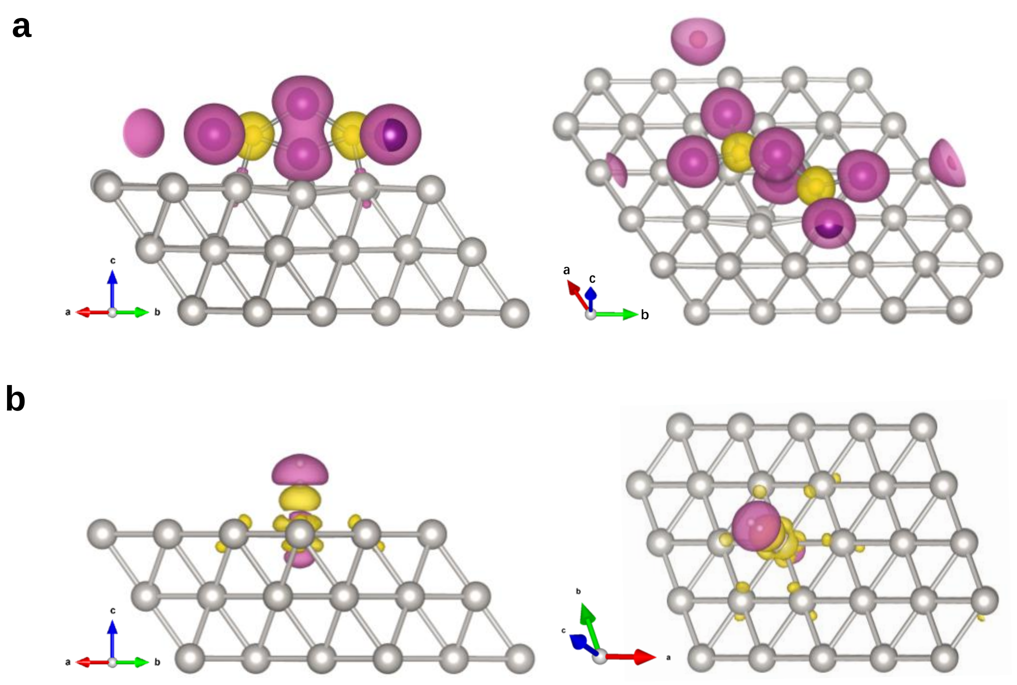

Fig. S29 The model used and the differential charge density of **a**, Li_6N_2 and **b**, H_2 on Pt (111).

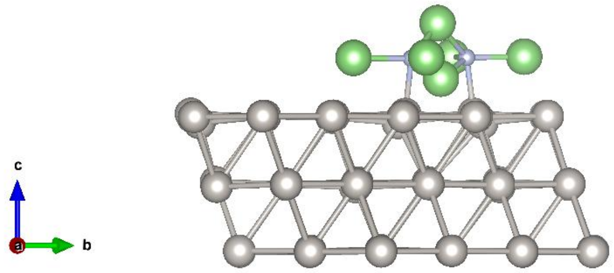

Fig. S30 The theoretical model of Li_6N_2@Pt(111) for differential charge density.

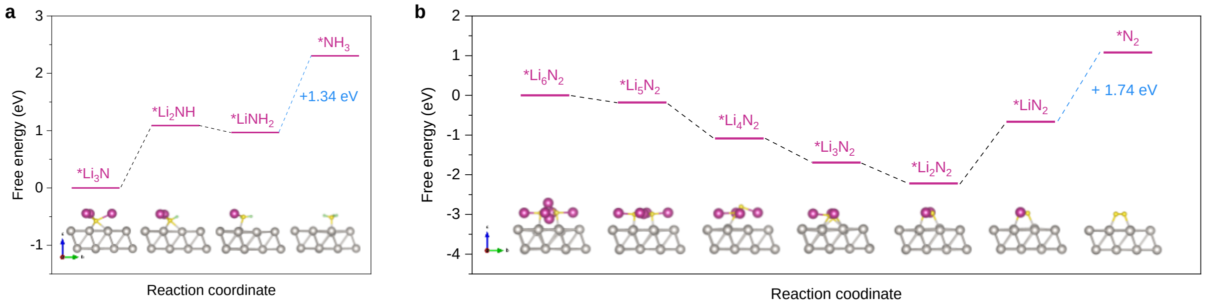

Fig. S31 a, Schematic Gibbs free energy diagrams of Li_3N protonation to NH_3 on Pt(111). **b**, Schematic Gibbs free energy diagrams of Li_6N_2 decomposition to N_2 on Pt (111).

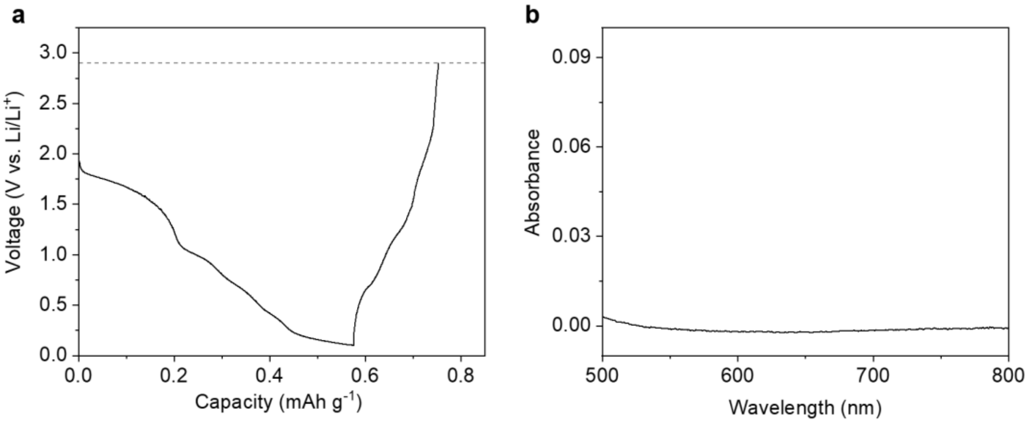

Fig. S32 **a**, Discharge-charge profile of Li-N_2/H_2 battery. **b**, Quantification of NH_3 via colorimetric method after charging the battery to 2.9 V in a H_2 atmosphere.

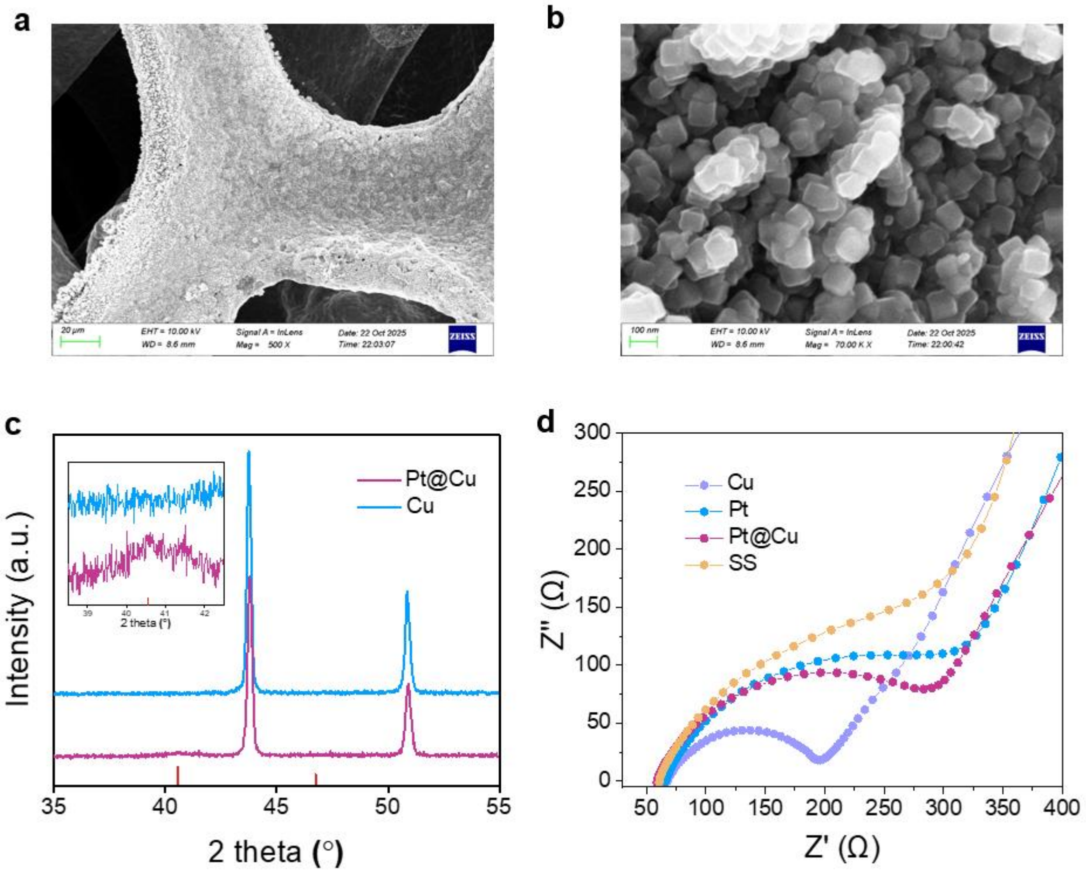

Fig. S33 Microstructural characterization of Pt/Cu electrode. **a,b,** SEM imaging of the Pt-electrodeposited copper foam clearly reveals the presence of cubic-shaped deposits uniformly distributed on the Cu skeleton. **c,** XRD analysis further confirms that these deposits correspond to metallic Pt, as indicated by the diffraction peaks matching the standard Pt reference. **d,** The Pt/Cu electrode exhibits a charge transfer resistance (*R*) of 140.08 Ω, which is significantly lower than that of pure Pt (275.70 Ω) and SS mesh (415.82 Ω). This notable reduction in *R* can be attributed to the three-dimensional porous architecture of the Cu foam, which offers a high specific surface area, enhances electrolyte accessibility, and facilitates the diffusion of reactants and products, thereby improving mass transport and overall electrochemical performance.

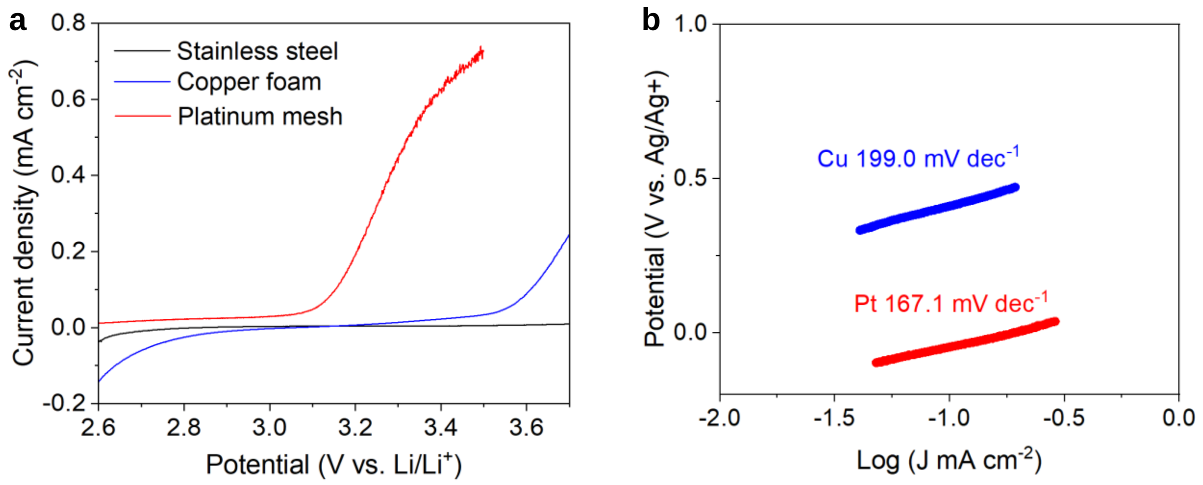

Fig. S34 a, LSV curves of Pt, copper foam and stainless-steel under a H_2 atmosphere. **b**, Tafel slope of Pt and Cu foam for the HOR.

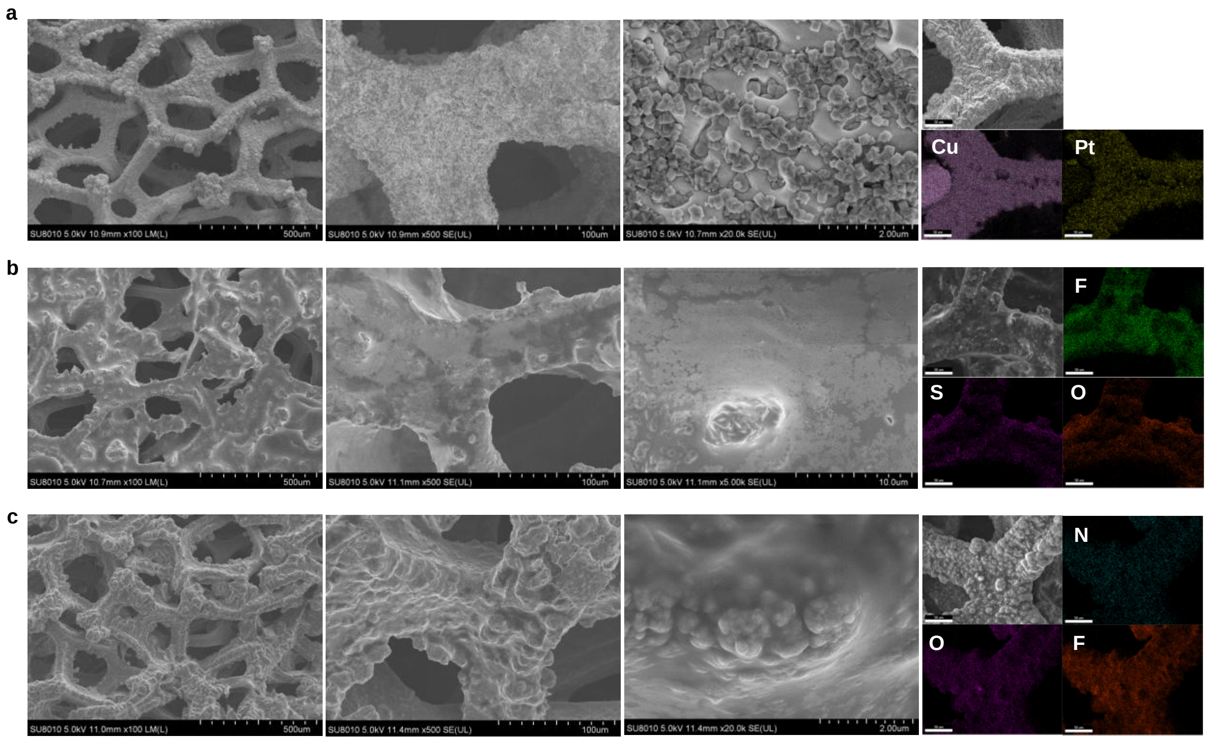

Fig. S35 Morphology and surface products characterization. **a**, SEM and EDS images of the pristine Pt/Cu working electrode. **b**, SEM and EDS images of Pt/Cu after discharging in Ar. The presence of F, O, S elements should be attributed to the reduction of electrolyte. **c**, SEM and EDS images of Pt/Cu after discharging in N_2. Elemental N was appeared after discharging in N_2, demonstrating successful catalytic N_2 reduction.

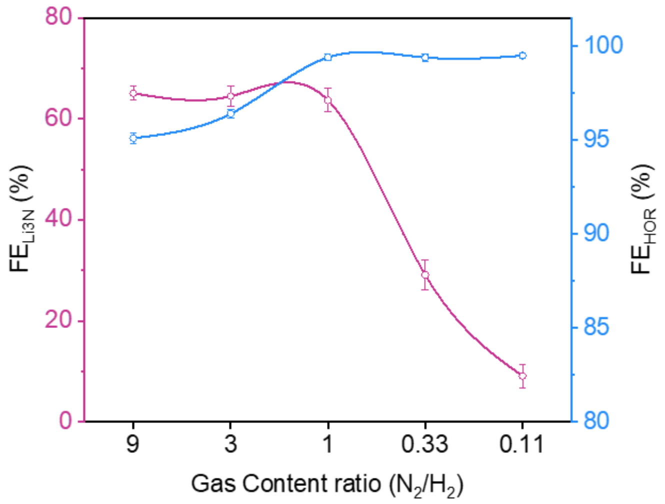

Fig. S36 Effect of partial pressure of H_2 on reaction efficiency. At a N_2/H_2 ratio of 9:1, the FE for Li_3N formation reached its highest value (65.1%), while the FE for HOR was 95.1%. As the H_2 partial pressure increased, the HOR FE progressively improved, approaching 100% at a 1:9 ratio. However, this enhancement in HOR came at the expense of Li_3N formation efficiency, which dropped sharply to 29.1% and 9.1% at N_2/H_2 ratios of 1:3 and 1:9, respectively.

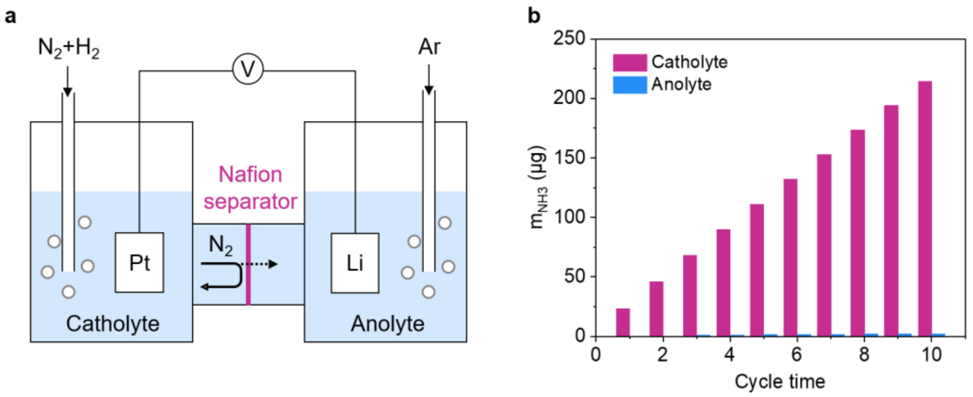

Fig. S37 H-type Li-N_2/H_2 battery. a, Schematic diagram of the assembly structure of the H-type Li-N_2/H_2 battery. **b**, The relationship between the amount of NH_3 produced in the catholyte and anolyte and the number of charge-discharge cycles. The catholyte was N_2/H_2-saturated LiTFSI in TEGDME, while the anolyte was Ar-saturated, with a Nafion membrane separating the two. The Nafion membrane, with its low N_2 permeability, prevented the diffusion of N_2 into the anolyte. Continuous Ar flow also removed any N_2 in the anolyte, preventing its contact with Li_0 anode. After 10 charge-discharge cycles, the NH_3 dissolved in the catholyte increased with the number of cycles, while only a minimal amount was detected in the anolyte, accounting for 1% of the total.

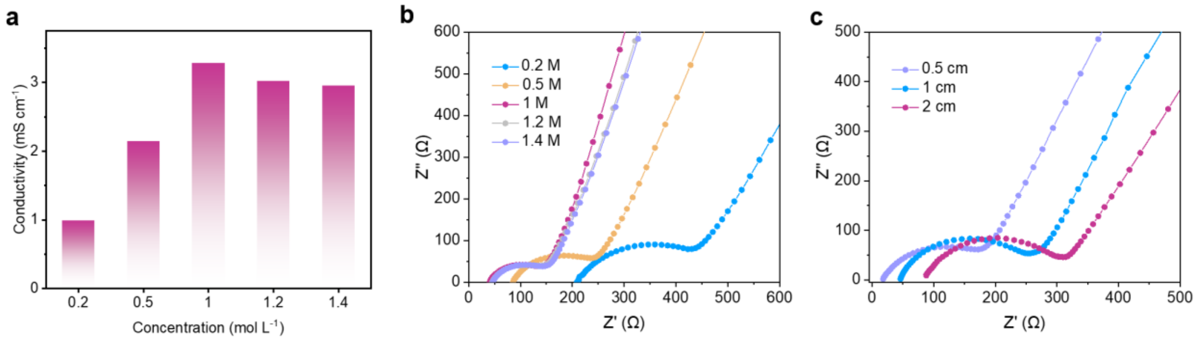

Fig. S38 Main contributors to the internal resistance analyzed by EIS plot. **a,** Ionic conductivity as a function of electrolyte concentration. **b,c,** EIS plots demonstrating the effect of electrolyte concentration (b) and electrode spacing (c).

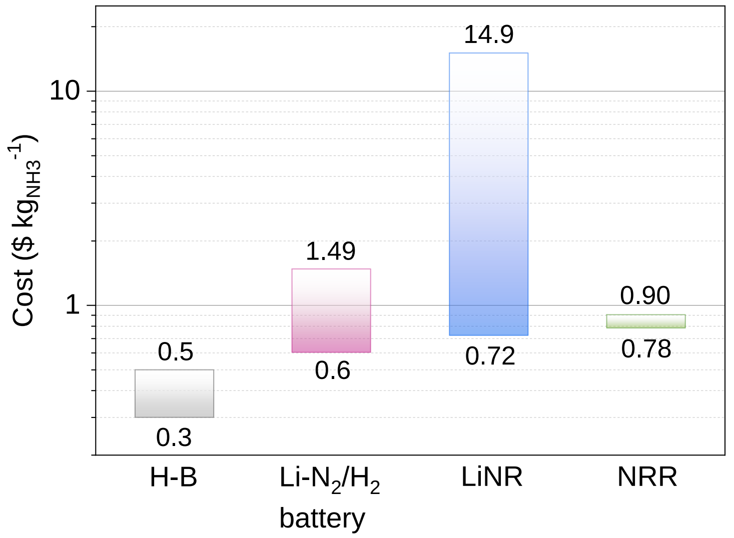

Fig. S39 Comparison of estimated cost of NH_3 production per kilogram for different methods. The maximum and minimum values of prices are calculated from Table S3, assuming the electricity price is \$0.03 kwh^{-1}.

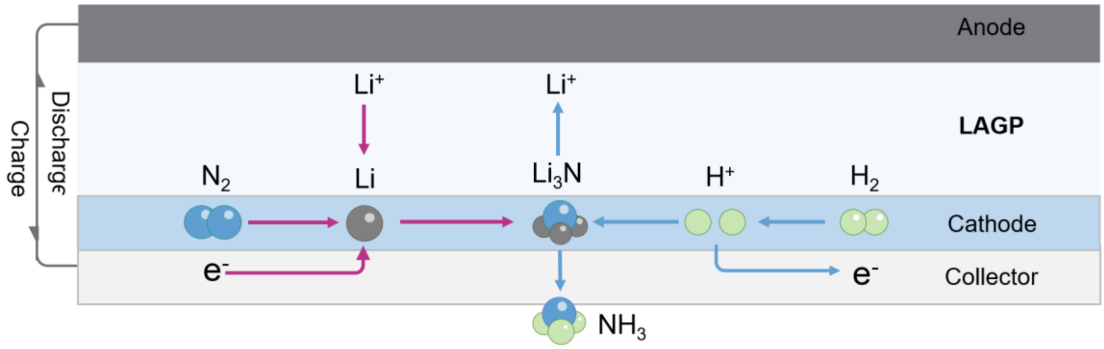

Fig. S40 Illustration of solid-state Li-N_2/H_2 battery.

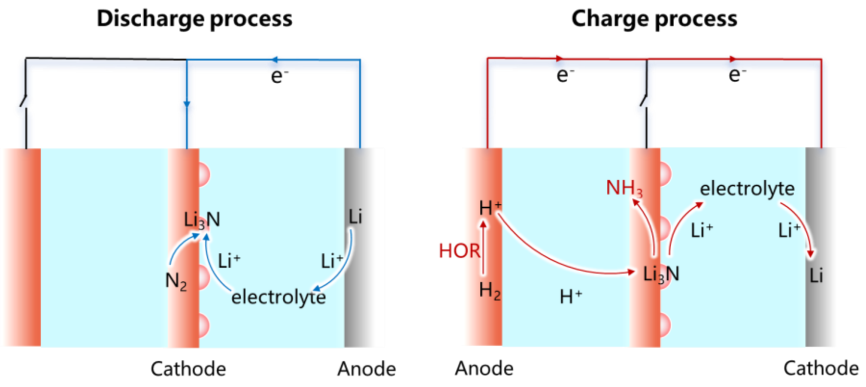

Fig. S41 Illustration of three-electrode Li-N_2/H_2 battery.: a lithium foil serving as the Li^+ storage/release electrode, while Pt@Cu-1 and Pt@Cu-2 electrodes are dedicated to NRR and HOR, respectively. During discharge, the lithium foil anode undergoes oxidation ($Li \rightarrow Li^+ + e^-$), and Li^+ migrates to the Pt@Cu-1 cathode for NRR to form Li_3N. During charging, the lithium foil acts as the cathode for Li^+ reduction ($Li^+ + e^- \rightarrow Li$), while the Pt@Cu-2 anode catalyzes HOR under H_2 atmosphere ($H_2 \rightarrow 2H^+ + 2e^-$). The resulting protons migrate through the electrolyte and react with Li_3N deposited on Pt@Cu-1 to produce NH_3. This three-electrode Li-N_2/H_2 battery system spatially separates the nitrogen reduction reaction and hydrogen oxidation reaction sites into independent electrodes, extending the "job-sharing effect" to the macroscopic scale, fundamentally avoiding active site blockage while maintaining high catalytic efficiency.

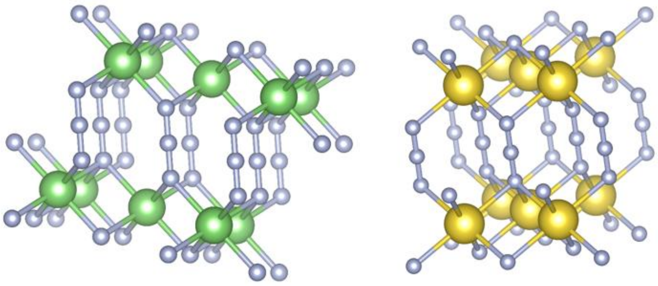

2Li⁺+3N₂+2e⁻=2LiN₃			
Energy	Li	N_2	LiN_3
$E_{1(KJ/mol)}$	-19970.45231	-287627.313	-451384.0669
$E_{(298.15\ K)}$	-------	-35.497056	58.13668
$E_{2(KJ/mol)}$	-19970.45231	-287662.8101	-451325.9302
$2Na^+ + 3N_2 + 2e^- = 2NaN_3$			
Energy	Na	N_2	NaN_3
$E_{1(KJ/mol)}$	-426378.6393	-287627.313	-857760.1944
$E_{(298.15\ K)}$	-------	-35.497056	49.526008
$E_{2(KJ/mol)}$	-426378.6393	-287662.8101	-857710.6684

Fig. S42 The theoretical physical models of LiN_3 (left) and NaN_3 (right) with the calculated energies. Notably, these formation potentials are significantly higher (less negative) than that of Li_3N [E(Li_3N) = –2.51 V vs. NHE for $3Li^+ + N_2 + 3e^- \rightarrow Li_3N$]. This shift effectively bridges the performance gap in the range of –2.5 V to –1.0 V vs. Li/Li^+ (converted from NHE), reducing the required overpotential and thereby improving energy efficiency. These theoretical results provide a reliable thermodynamic basis for addressing the performance gap in the target overpotential window and guide the rational design of highly efficient ammonia electrosynthesis systems.

Supplementary Tables

Table S1. Summary of gas purity analysis.

Gas	Ar	$^{14}N_2$	$^{15}N_2$
Purity	99.999%	99.999%	99%
NH_3 (ppb)	/	2.26 ±0.02 [a]<br>2.21 ±0.01 [b]	/

[a] Quantified by colorimetric measurement

[b] Quantified by ^{1}H NMR

"/" represents negative value which is meaningless when calculating impurities amount

Table S2. Summary of the quantification of N-impurities of the electrolyte, chemicals and glassware.

Impurities	Electrolyte	Chemicals (mainly Li-salts)	Glassware
NO_2^-, NO_3^- (ppb)	/	/	/
NH_3 (ppb)	/	1.14 ±0.03 [a]<br>1.38 ±0.02 [b]	/

[a] Quantified by colorimetric measurement

[b] Quantified by ^{1}H NMR

"/" represents negative value which is meaningless when calculating impurities amount

Table S3. Performance comparison.

Electrode	WE voltage (V vs $Li^{0/+}$)	FE (%)	NH_3 production rate			EE (%)	Ref.
			nmol s^{-1} mA^{-1}	μg mg_{cat}^{-1} h^{-1}	nmol s^{-1} cm^{-2}		
HBTCu	-1	13.3	0.23	-	46	2.3	[11]
Au/CP	-1	34	1.6	-	0.8	7	[12]
MEA	-0.25	8.9	0.13	-	0.67	2	[13]
Mo	-1	37	0.35	-	0.7	7	[14]
Ni	-0.55	86	2.8	-	230	12.3	[15]
Cu	-2	18.5	1	-	7.9	1.45	[16]
Cu	-2	71	2.5	-	2500	7.7	[17]
SS	-1	35	1.2	-	30	2.8	[18]
Ni	-0.55	91	4.1	-	150	15	[19]
SS	-0.66	61	2.1	-	0.5	13	[20]
Mo	-1.5	78	2.7	-	10.8	11.7	[21]
Cu	-1	69	1.76	-	53	13	[22]
CC	1	59	2	-	0.1	-	[23]
Graphene	1	10	-	-	0.01	-	[24]
B-graphene	2.6	10.8	0.4	-	0.16	7.6	[25]
IrTe	2.9	11.2	-	34.6	-	9	[26]
Ru@ZrO_2/NC	2.82	21	-	3.66	1.1	17	[27]
Pd_3Bi	2.78	21	-	59	1.9	16	[28]
Au_4Pt_2/G	2.78	12	-	27.1	0.11	9.4	[29]
TiO_2/CC	2.68	32.1	-	1.8	0.3	23.5	[30]
Mo/VO_2	2.48	32.4	-	190.1	1.5	21.1	[31]
MoO_{3-x}/MXene	2.68	22.3	-	95.8	0.16	16.4	[32]
Fe_{SA}-NO-C	2.58	11.8	-	31.9	0.13	8.1	[33]
Ru SAs/GDY/G	2.27	32.4	-	60.1	0.49	18.9	[34]
c-COFs	2.82	31.9	-	33.6	0.55	25.7	[35]
Pt/Cu	0.05~0.5	65	2.2	100	3.5	26	This work

Supplementary Note 1: Electrocatalytic nitrogen fixation activity of Pt

A comprehensive mechanistic study employing a suite of characterization techniques, including electrochemistry, spectroscopy, and theoretical modeling. Our integrated analysis demonstrates that lithium nitride (Li_3N) formation proceeds predominantly through a direct electrocatalytic pathway ($N_2 + 6e^- + 6Li^+ \rightarrow 2Li_3N$), rather than an indirect thermochemical route involving metallic lithium ($Li^+ + e^- \rightarrow Li$, $6Li + N_2 \rightarrow 2Li_3N$). The key evidence is summarized as follows:

1. Electrochemical evidence for the alloying mechanism: LSV measurements in an Ar atmosphere reveal that the reduction current on both Cu and Pt electrodes originates from a lithium alloying process, rather than lithium metal deposition. The difference in current response is attributed to their distinct lithiophilicity: the relatively lithiophobic nature of Cu results in lower alloying capacity and a smaller reduction current compared to Pt. To further verify that the process occurring on Pt near 0 V vs. Li/Li^+ corresponds to alloying rather than Li plating, we performed ex situ XRD on the discharged electrode (Fig. S43). The observed peak broadening and shifts toward lower angles are consistent with lithium intercalation into the metallic lattice and the accompanying expansion of lattice constants—a hallmark of alloy formation. Importantly, no diffraction signals corresponding to metallic Li were detected.

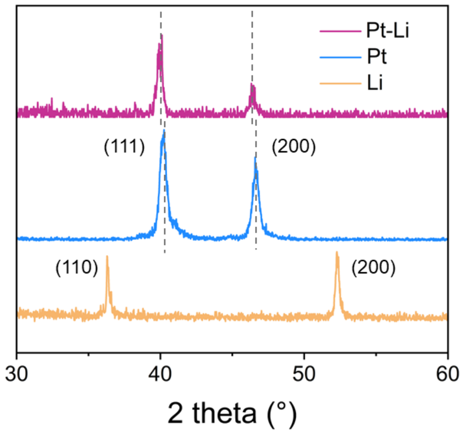

Fig. S43 XRD patterns of electrode before/after discharged in Ar. The strong peaks at 41 ° and 46.5 ° in the pristine Pt correspond to the (111) and (200) crystal planes, respectively. After discharge in an Ar atmosphere, the resulting Pt-Li exhibits clear peak broadening and a distinct shift toward lower angles. These changes are consistent with the intercalation of lithium into the metal lattice and the accompanying expansion of the lattice constant. Furthermore, no diffraction peaks corresponding to metallic lithium (e.g., at 37 ° for the (110) plane or 52.5 ° for the (200) plane) are observed, confirming the absence of significant metallic lithium in the discharge product.

2. XPS analysis confirming the absence of metallic Li: XPS depth profiling on the Pt electrode discharged in Ar (Fig. S44) further confirms that no appreciable metallic Li^0 accumulates within the operational potential window (>0 V vs. Li/Li^+). The near-surface region is dominated by SEI components (e.g., Li–F and Li–O species), while deeper regions reveal Li–Pt alloy and metallic Pt, with no spectral evidence of Li^0. This result aligns with our earlier report on Li–N_2 batteries (*Nat. Catal.* 2023, 7, 55–64), in which a Ni-based electrocatalyst under N_2 formed Li_3N and SEI without detectable Li metal.

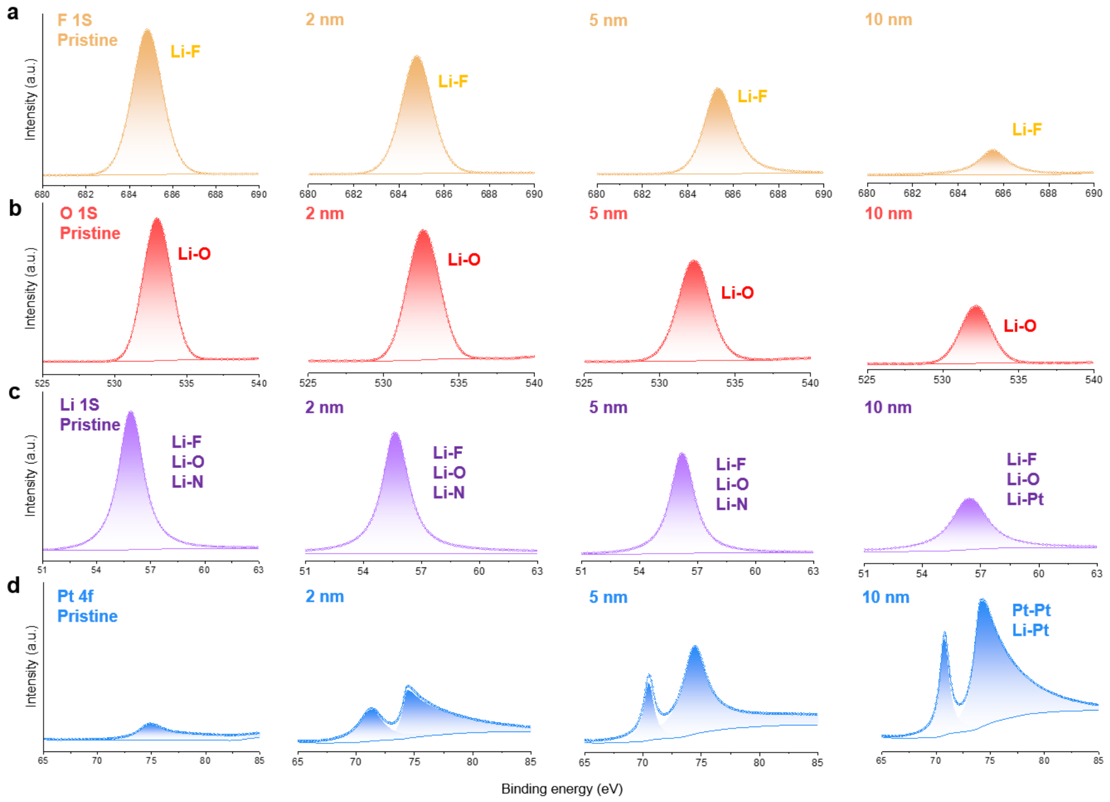

Fig. S44 XPS depth profiling spectra within 0-10 nm for Pt electrode being discharged in Ar atmosphere. (a) F 1s, (b) O 1s, (c) Li 1s and (d) Pt 4f. Rate of etching, ~1 nm s^{-1}.

3. Our DFT calculations reveal the fundamental origins of the superior catalytic capability of Pt over Cu. Analysis of the N_2 adsorption configurations (Fig. S45) shows an end-on binding mode on both surfaces. Notably, the N–N bond length is significantly elongated on Pt (1.13 Å) compared to on Cu (1.11 Å), indicating more effective activation of the N≡N bond on Pt. The electronic interactions underlying this activation were probed by projected density of states (PDOS) analysis. The PDOS profiles (Fig. S45) demonstrate a markedly stronger hybridization between N_2-derived states and Pt orbitals than with Cu orbitals, correlating with a higher N_2 binding energy on Pt. Furthermore, the *d*-band center of the Pt–N_2 system is closer to the Fermi level than that of Cu–N_2, signifying that the *d*-electrons of Pt are more readily available to participate in the nitrogen reduction reaction.

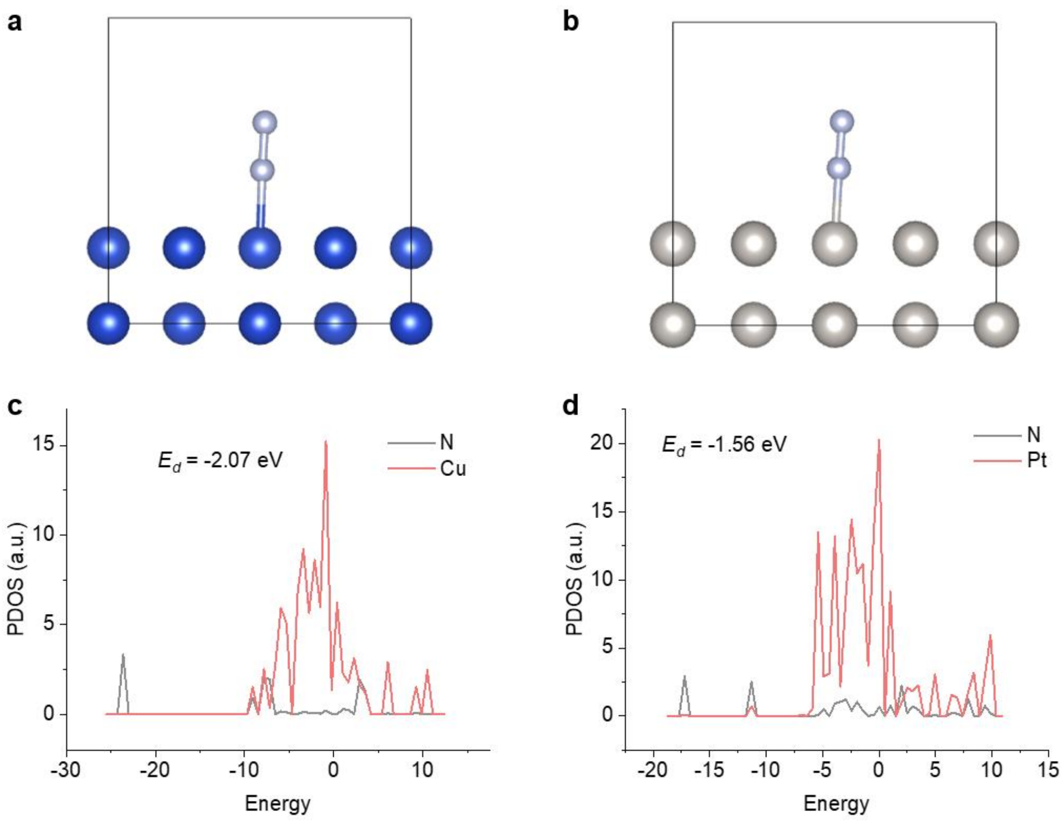

Fig. S45 Optimized structures of N_2 adsorbed on (a) Cu(111) and (b) Pt(111). (c,d) The corresponding projected density of states (PDOS) profiles.

We further propose a plausible electrocatalytic pathway involving the sequential addition of Li^+ ions and electrons to adsorbed N_2 (Fig. 46). The free energy diagram shows that, except for the rate-determining step (RDS) involving the transition from $*Li_2N_2$ to *N and subsequent Li_3N desorption, all other intermediate steps are exothermic on Pt. The lower free energy barrier for this RDS on Pt (ΔG = +2.94 eV) compared to Cu (ΔG = +3.52 eV) provides a quantitative explanation for the enhanced catalytic performance of Pt. This proposed mechanism is consistent with previous studies on Li–N_2 and Zn–N_2 batteries, lending further credibility to our analysis (*Adv. Energy Mater.* 2023, 13, 2300269; *J. Mater. Chem. A* 2019, 7, 19950-19960).

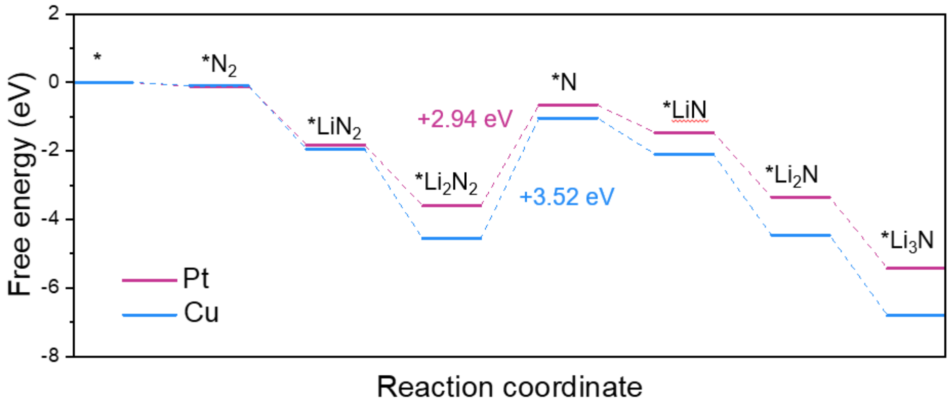

Fig. S46 Schematic Gibbs free energy diagrams. Except for the rate-determining step, i.e., the transition reaction from $*Li_2N_2$ to *N by the addition of one Li^+ and e^- and the desorption of one Li_3N molecule, the rest of the reaction is exothermic. Pt exhibits a lower free energy (+2.94 eV) for the rate-determining step, which accounts for the improved catalytic activity. In contrast, the free energy for the rate-determining step is +3.52 eV on Cu surface, further indicating its limited catalytic performances.

To elucidate the reaction mechanism, we conducted DFT calculations to explore two distinct N_2 adsorption modes on the Pt(111) surface. As shown in Fig. S47a and b, N_2 can adsorb in either an end-on or a side-on configuration. The side-on adsorption mode leads to significant elongation of the N≡N bond length (1.31 Å vs. 1.18 Å in the end-on mode), indicating more effective activation of the dinitrogen molecule. The calculated free-energy profiles (Fig. S47c) reveal that the end-on pathway exhibits a prohibitively high energy barrier of 2.94 eV for the conversion from Pt–Li_2N_2 to Pt–N + Li_3N. In sharp contrast, the side-on pathway presents a markedly lower barrier of only 0.48 eV, suggesting that this route is kinetically favorable under experimental conditions. These results strongly indicate that the side-on adsorption configuration is the dominant mechanism in our catalytic system. Further structural analysis of the key intermediate states along the side-on pathway (Fig. S47d) illustrates the progressive weakening and cleavage of the N≡N bond, facilitated by lithium coordination, ultimately leading to the formation of Li_3N.

Overall, we have demonstrated the decisive impact of N_2 adsorption strength, electronic interactions and rate-determining step energy (all being pivotal catalysis-related determinants), on the N_2 reduction performances of the selected metal. These results support our hypothesis that the N_2 reduction to Li_3N in our work is very likely an electrocatalytic process. The source of Li_3N is currently the subject of active research, with both thermochemical Li mediation and electrocatalysis as possible mechanisms as summarized by Prof. Karthish Manthiram (*Nat. Energy* 2023, 8, 138–148). Since our working potential (>0 V vs. Li/Li^+) constrains Li metal deposition at the cathode, the formation of Li_3N through

thermochemical reactions is expected to be limited. We sincerely hope the reviewer will find our data supportive of our conclusions.

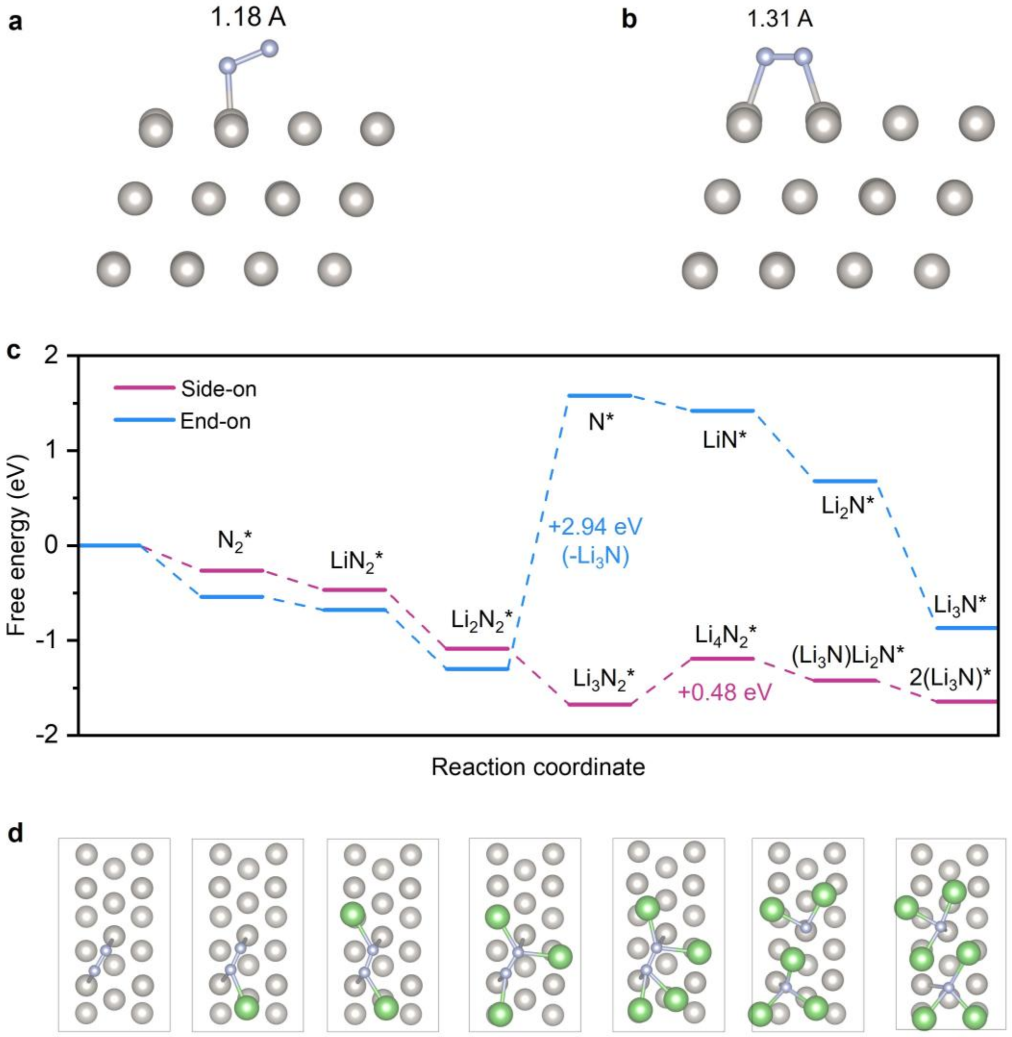

Fig. S47 a, End-on and **b**, side-on adsorption modes of N_2 molecule on Pt(111) surface. **c**, Calculated energy profiles for electrocatalytic N_2 reduction to Li_3N via end-on and side-on adsorption modes. d, Structural models of intermediate states along the side-on adsorption pathway.

Supplementary Note 2: Key aspects of the bifunctional catalytic electrode

1. Bifunctional catalysis: The central function of the Pt electrode is its ability to independently and efficiently catalyze two distinct reactions: N_2 reduction to Li_3N during discharge, and H_2 oxidation during charge. DFT calculations reveal that these reactions preferentially occur at different types of active sites on the Pt surface. This intrinsic "job-sharing" effect allows the two catalytic cycles to be cascaded on a single electrode without mutual interference

2. Kinetic prioritization: A key operational feature of the electrode is its ability to kinetically prioritize the HOR over Li_3N oxidation during charging. Experimental results confirm that the presence of H_2 substantially lowers the charging potential. The underlying reason, supported by theoretical analysis, is that the energy barrier for Li_3N protonation is significantly lower than that for its direct oxidation. Thus, the Pt electrode actively creates a thermodynamic preference for the NH_3 synthesis pathway when H_2 is present.

3. Distinct reaction route: Critically, the bifunctional electrocatalytic nature of the Pt electrode fundamentally distinguishes our coupled nitrogen reduction–protonation pathway from conventional lithium-mediated routes. Unlike mechanisms that rely on the electrochemical deposition of metallic lithium followed by a thermochemical reaction with N_2, our system enables a direct electrocatalytic conversion of N_2 to Li_3N without generating bulk metallic Li. This pivotal distinction decouples electron transfer for N_2 activation from proton donation, thereby circumventing the high overpotentials and hydrogen evolution competition inherent in metallic lithium formation.

In summary, the Pt electrode functions not as a simple conductor, but as an integrated bifunctional catalyst and reaction platform. It orchestrates the entire synthetic cycle by: (i) providing distinct active sites for N_2 reduction and H_2 oxidation, (ii) kinetically steering the reaction toward NH_3 production, and (iii) maintaining the stability of the Li_3N intermediate until protonation can occur. We have revised the manuscript to foreground these sophisticated roles and believe this mechanistic perspective significantly strengthens the narrative of our work.

Supplementary Note 3: Estimation of cost

We calculated the total production cost of the Li-N_2/H_2 battery, estimated as the sum of the operational and capital expenditures (OPEX and CAPEX, respectively).

CAPEX

The primary component of CAPEX includes the cost of hydrogen and the Pt-based catalyst. Assuming 100% efficiency for the hydrogen oxidation and protonation reactions, synthesizing 1 kg of NH_3 requires at least 176 g of hydrogen. Based on the 2024 cost of electrolytic hydrogen production at \$1.4 per kg of H_2, the hydrogen feedstock cost is estimated at \$0.25 kg_{NH3}^{-1}. Regarding the Pt catalyst, assuming that a loading of 2 mg cm^{-2} was applied to the electrode. Under a current density of 100 mA cm^{-2} and a Faradaic efficiency of 21.54% for NRR, the catalyst-related cost per kg of NH_3 is approximately \$1.02, based on the 2024 Pt price (~\$30 per gram). We also evaluated the impact of catalyst lifespan: assuming the catalyst retains 80% of its initial activity after 2,000 hours of operation, the amortized Pt cost per kg of NH_3 becomes ~\$1.28. This highlights the importance of developing low-Pt or Pt-free catalysts for long-term economic feasibility.

OPEX

Operational costs include device components and balance of plant (BoP). For a 1 m^2 battery operating at 100 mA cm^{-2} for 10 hours, kg-scale NH_3 production is achievable (assuming 60% NRR efficiency and 100% HOR efficiency). Key components—lithium foil, current collector, and electrolyte—total ~\$190. Assuming a device lifetime of 2,000 hours (based on Li–O_2 battery benchmarks) and a production of 200 kg NH_3 before replacement, the component cost is ~\$0.95 kg_{NH3}^{-1}.

BoP costs, estimated at 50% of system cost under long-term room-temperature N_2/H_2O operation (including recycling and purification subsystems), add ~\$0.48 kg_{NH3}^{-1}. Electricity cost, as noted in the main text, ranges from \$0.6–\$1.49 kg_{NH3}^{-1} depending on local green electricity prices and system Faradaic efficiency.

Total Cost

Incorporating all factors, the total estimated cost for 1 kg of NH_3 is approximately \$2.28–\$3.17 kg_{NH3}^{-1}, with Pt catalyst cost and stability representing a major influence on CAPEX. These values are subject to variation based on regional material costs and operational conditions. All unit prices reflect 2024 levels in China.

References

1. Lazouski N, Limaye A, Bose A *et al.* Cost and performance targets for fully electrochemical ammonia production under flexible operation. *ACS Energy Lett.* 2022; **7**(8): 2627-2633.
2. Wang M, Khan MA, Mohsin I *et al.* Can sustainable ammonia synthesis pathways compete with fossil-fuel based Haber–Bosch processes? *Energy Environ. Sci.* 2021; **14**(5): 2535-2548.
3. MacFarlane DR, Cherepanov PV, Choi J *et al.* A roadmap to the ammonia economy. *Joule* 2020; **4**(6): 1186-1205.
4. Ye D, Tsang SCE. Prospects and challenges of green ammonia synthesis. *Nat. Synth.* 2023; **2**: 612-623.
5. Zhang X, Xiong W, Wang T *et al.* Cascade electrosynthesis of LiTFSI and N-containing analogues via a looped Li–N_2 battery. *Nat. Catal.* 2024; **7**: 55-64.
6. Kresse G, Furthmuller J. Efficient iterative schemes for ab initio total-energy calculations using a plane-wave basis set. *Phys. Rev. B Condens Matter* 1996; **54**(16): 11169-11186.
7. Perdew JP, Burke K, Ernzerhof M. Generalized Gradient Approximation Made Simple. *Phy.s Rev. Lett.* 1996; **77**(18): 3865-3868.
8. Blochl PE. Projector augmented-wave method. *Phys. Rev. B Condens Matter* 1994; **50**(24): 17953-17979.
9. Grimme S, Antony J, Ehrlich S *et al.* A consistent and accurate ab initio parametrization of density functional dispersion correction (DFT-D) for the 94 elements H-Pu. *J. Chem. Phys.* 2010; **132**(15): 154104.
10. Skulason E, Bligaard T, Gudmundsdottir S *et al.* A theoretical evaluation of possible transition metal electro-catalysts for N_2 reduction. *Phys. Chem. Chem. Phys.* 2012; **14**(3): 1235-1245.
11. Li K, Shapel SG, Hochfilzer D *et al.* Increasing current density of Li-mediated ammonia synthesis with high surface area copper electrodes. *ACS Energy Lett.* 2021; **7**(1): 36-41.
12. Gao LF, Cao Y, Wang C *et al.* Domino effect: Gold electrocatalyzing lithium reduction to accelerate nitrogen fixation. *Angew. Chem. Int. Ed. Engl.* 2021; **60**(10): 5257-5261.
13. Cai X, Shadike Z, Cai X *et al.* Membrane electrode assembly design for lithium-mediated electrochemical nitrogen reduction. *Energy Environ. Sci.* 2023; **16**: 3063-3073.
14. Andersen SZ, Statt MJ, Bukas VJ *et al.* Increasing stability, efficiency, and fundamental understanding of lithium-mediated electrochemical nitrogen reduction. *Energy Environ. Sci.* 2020; **13**(11): 4291-4300.
15. Du H-L, Matuszek K, Hodgetts R *et al.* The chemistry of proton carriers in high-performance lithium mediated ammonia electrosynthesis. *Energy Environ. Sci.* 2023; **16**: 1082–1090.
16. Lazouski N, Schiffer ZJ, Williams K *et al.* Understanding continuous lithium-mediated electrochemical nitrogen reduction. *Joule* 2019; **3**: 1127-1139.
17. Li S, Zhou Y, Li K *et al.* Electrosynthesis of ammonia with high selectivity and high rates via engineering of the solid-electrolyte interphase. *Joule* 2022; **6**: 1-19.
18. Lazouski N, Chung M, Williams K *et al.* Non-aqueous gas diffusion electrodes for rapid ammonia synthesis from nitrogen and water-splitting-derived hydrogen. *Nat. Catal.* 2020; **3**(5): 463-469.
19. Du HL, Chatti M, Hodgetts RY *et al.* Electroreduction of nitrogen with almost 100% current-to-ammonia efficiency. *Nature* 2022; **609**(7928): 722-727.
20. Fu X, Pedersen JB, Zhou Y *et al.* Continuous-flow electrosynthesis of ammonia by nitrogen reduction and hydrogen oxidation. *Science* 2023; **379**: 707–712.
21. Li K, Andersen SZ, Statt MJ *et al.* Enhancement of lithium-mediated ammonia synthesis by addition of oxygen. *Science* 2021; **374**: 1593–1597
22. Suryanto BHR, Matuszek K, Choi J *et al.* Nitrogen reduction to ammonia at high efficiency and rates based on a phosphonium proton shuttle. *Science* 2021; **372**: 1187–1191.
23. Ma J, Bao D, Shi M *et al.* Reversible nitrogen fixation based on a rechargeable lithium-nitrogen battery for energy storage. *Chem* 2017; **2**(4): 525-532.
24. Zhang Z, Wu S, Yang C *et al.* Li-N_2 batteries: A reversible energy storage system? *Angew. Chem. Int. Ed. Engl.* 2019; **58**(49): 17782-17787.

25. Yu X, Han P, Wei Z *et al.* Boron-doped graphene for electrocatalytic N_2 reduction. *Joule* 2018; **2**(8): 1610-1622.
26. Jiang B, Xue H, Wang P *et al.* Noble-metal-metalloid alloy architectures: Mesoporous amorphous iridium-tellurium alloy for electrochemical N_2 reduction. *J. Am. Chem. Soc.* 2023; **145**(11): 6079-6086.
27. Tao H, Choi C, Ding L-X *et al.* Nitrogen fixation by Ru single-atom electrocatalytic reduction. *Chem* 2019; **5**(1): 204-214.
28. Wang X, Luo M, Lan J *et al.* Nanoporous intermetallic Pd_3Bi for efficient electrochemical nitrogen reduction. *Adv. Mater.* 2021; **33**(18): e2007733.
29. Yao C, Guo N, Xi S *et al.* Atomically-precise dopant-controlled single cluster catalysis for electrochemical nitrogen reduction. *Nat. Commun.* 2020; **11**(1): 4389.
30. Guo Y, Gu J, Zhang R *et al.* Molecular crowding effect in aqueous electrolytes to suppress hydrogen reduction reaction and enhance electrochemical nitrogen reduction. *Adv. Energy Mater.* 2021; **11**(36): 2101699.
31. Xie M, Dai F, Guo H *et al.* Improving electrocatalytic nitrogen reduction selectivity and yield by suppressing hydrogen evolution reaction via electronic metal-support interaction. *Adv. Energy Mater.* 2023; **13**(21): 2203032.
32. Chu K, Luo Y, Shen P *et al.* Unveiling the synergy of O-vacancy and heterostructure over MoO_3-x/MXene for N_2 electroreduction to NH_3. *Adv. Energy Mater.* 2021; **12**(3): 2103022.
33. Li Y, Li J, Huang J *et al.* Boosting electroreduction kinetics of nitrogen to ammonia via tuning electron distribution of single-atomic iron sites. *Angew. Chem. Int. Ed. Engl.* 2021; **60**(16): 9078-9085.
34. Feng X, Liu J, Chen L *et al.* Hydrogen radical-induced electrocatalytic N_2 reduction at a low potential. *J. Am. Chem. Soc.* 2023; **145**(18): 10259-10267.
35. Zhong H, Wang M, Ghorbani-Asl M *et al.* Boosting the electrocatalytic conversion of nitrogen to ammonia on metal-phthalocyanine-based two-dimensional conjugated covalent organic frameworks. *J. Am. Chem. Soc.* 2021; **143**(47): 19992-20000.
